# Antiageing strategy for neurodegenerative diseases: from mechanisms to clinical advances

**DOI:** 10.1038/s41392-025-02145-7

**Published:** 2025-03-10

**Authors:** Qiu Jiang, Jie Liu, Shan Huang, Xuan-Yue Wang, Xiaowei Chen, Guang-Hui Liu, Keqiang Ye, Weihong Song, Colin L. Masters, Jun Wang, Yan-Jiang Wang

**Affiliations:** 1 Department of Neurology and Centre for Clinical Neuroscience, Daping Hospital, Third Military Medical University, Chongqing, China; 2 Chongqing Key Laboratory of Ageing and Brain Diseases, Chongqing, China; 3 Chongqing Institute for Brain and Intelligence, Guangyang Bay Laboratory, Chongqing, China; 4 Brain Research Center, Third Military Medical University, Chongqing, China; 5 University of Chinese Academy of Sciences, Beijing, China; 6 State Key Laboratory of Membrane Biology, Institute of Zoology, Chinese Academy of Sciences, Beijing, China; 7 Faculty of Life and Health Sciences, and Brain Cognition and Brain Disease Institute (BCBDI), Shenzhen Institute of Advanced Technology, Chinese Academy of Sciences, Shenzhen, China; 8 Institute of Aging, Key Laboratory of Alzheimer’s Disease of Zhejiang Province. Zhejiang Clinical Research Center for Mental Disorders, School of Mental Health and The Affiliated Kangning Hospital, Oujiang Laboratory (Zhejiang Lab for Regenerative Medicine, Vision and Brain Health), Wenzhou Medical University, Wenzhou, Zhejiang China; 9 The Florey Institute, The University of Melbourne, Parkville, VIC Australia; 10 State Key Laboratory of Trauma and Chemical Poisoning, Chongqing, China

**Keywords:** Diseases of the nervous system, Cellular neuroscience, Molecular neuroscience

## Abstract

In the context of global ageing, the prevalence of neurodegenerative diseases and dementia, such as Alzheimer’s disease (AD), is increasing. However, the current symptomatic and disease-modifying therapies have achieved limited benefits for neurodegenerative diseases in clinical settings. Halting the progress of neurodegeneration and cognitive decline or even improving impaired cognition and function are the clinically meaningful goals of treatments for neurodegenerative diseases. Ageing is the primary risk factor for neurodegenerative diseases and their associated comorbidities, such as vascular pathologies, in elderly individuals. Thus, we aim to elucidate the role of ageing in neurodegenerative diseases from the perspective of a complex system, in which the brain is the core and peripheral organs and tissues form a holistic network to support brain functions. During ageing, the progressive deterioration of the structure and function of the entire body hampers its active and adaptive responses to various stimuli, thereby rendering individuals more vulnerable to neurodegenerative diseases. Consequently, we propose that the prevention and treatment of neurodegenerative diseases should be grounded in holistic antiageing and rejuvenation means complemented by interventions targeting disease-specific pathogenic events. This integrated approach is a promising strategy to effectively prevent, pause or slow down the progression of neurodegenerative diseases.

## Introduction

According to the 2022 World Health Organization (WHO) report, the speed of population ageing in countries around the world is far faster than that in the past, and the number and proportion of elderly individuals are on the rise. From 2020 to 2050, the global population aged 60 years and over is projected to increase from 1 billion to 2.1 billion, while the number of people aged 80 and over is expected to triple to 426 million. Due to the prosperous development of biomedicine, human life and life expectancy continue to rise worldwide, which is not consistent with the healthspan.^1^ The gap in lifespan and healthspan means that large numbers of older people are living with age-related diseases for long periods, imposing a substantial economic and caregiving burden on families and society. Disability-adjusted life years (DALYs) are proposed, including years lived with disability (YLDs) and years of life lost (YLLs), to quantify the burden caused by diseases. In 2021, for individuals aged 60–79 years, Alzheimer’s disease (AD) and other forms of dementia ranked second among the top three leading causes of DALYs, whereas Parkinson’s disease (PD) ranked third for those aged 80 years and older.^2^ Neurodegenerative diseases, including AD, PD, amyotrophic lateral sclerosis (ALS), frontotemporal lobar degeneration (FTLD), Huntington’s disease (HD) and others, are major diseases that cause dementia, disability, loss of independence and even death in elderly individuals. The incidence of neurodegenerative diseases is substantially increasing in the elderly population; dementia currently affects more than 55.2 million individuals worldwide, and this number is projected to reach 78 million by 2030 from the World Alzheimer Report 2021.^3^ AD is the most prevalent type of dementia, accounting for 60–80% of cases.^4^ PD is the second most common neurodegenerative disease, with a rapid increase in incidence after the age of 50 years. According to the 2019 WHO estimates, 850,000 people suffer from PD worldwide. In general, in the context of accelerated global ageing, the global burden of other neurodegenerative diseases, such as ALS, HD and FTLD, is increasing. Society bears a heavy burden of increasing neurodegenerative disease costs.^5^ For example, the global costs of dementia are expected to increase nearly tenfold to $9.12 trillion from 2015 to 2050,^6^ and a similar situation is predicted for other neurodegenerative diseases.^7,8^

The ageing process is accompanied by the accumulation of genetic mutations and epigenetic changes, which gradually disrupt functional homoeostasis at the molecular and cellular levels, leading to loss of proteostasis and abnormal mitochondrial function. In the context of neurodegenerative diseases, proteostasis loss substentially contributes to the abnormal accumulation of various pathological proteins, including amyloid-beta (Aβ), hyperphosphorylated tau, α-synuclein (α-syn), TAR DNA binding protein-43 (TDP-43), huntingtin (HTT). These aberrant proteins act as activators for glial cells, triggering neuroinflammation and other pathological events. Subsequent inflammation exerts detrimental effects on neurons, resulting in neuronal injury, disruption of neural circuitry, and eventual manifestation of diverse neurodegenerative disorders.

Regrettably, current therapeutics are severely limited, and no interventions are available to stop or even reverse the course of these diseases. The clinical interventions for PD, FTLD and HD have concentrated on symptomatic treatment and nonpharmacological approaches (e.g., lifestyle modifications, peer and caregiver support), and no efficacious drug has been demonstrated to have disease-modifying effects on patients.^9–11^ Although electroencephalogram-based brain‒computer interface (BCI) technology can help ALS patients communicate with the outside world via real-time speech synthesis and robotic arms,^12,13^ disease progression cannot be affected. For HD, clinical trials of drugs targeting proximal molecules, namely, HTT DNA, RNA and protein, are underway and may be available to modify the disease course in the future.^14^ Surprisingly, recent clinical trials of Aβ-targeted immunotherapies have shown their efficacy in slowing cognitive decline.^15^ Nevertheless, the overall cognitive benefits of these treatments are limited once the dementia stage has taken hold.^16–18^ These realities highlight the urgent need to explore more effective therapeutic strategies for ageing-related neurodegenerative diseases.

## Impact of ageing on neurodegenerative diseases

Ageing encompasses suborganismal biological processes leading to declines in organismal survival and function over time,^19^ which is the basis of many chronic diseases. The incidence of AD increases exponentially after the age of 65.^4^ Epidemiological studies have documented a significant increase in the percentage of individuals with AD with age, especially women, which were reported as early as 2000.^20^ In 2022 in the United States, the percentage of individuals with AD ranged from 5% among individuals aged 65–74 years, 13.1% among individuals aged 75–84 years, and 33.2% among individuals aged 85 years and above.^21^ The incidence of PD also increases with increasing age, and whether this association is linear or exponential is unclear.^22^ Based on the MEDLINE and EMBASE databases, an analysis was conducted to determine the global prevalence of PD between 1985 and 2010 across different age groups. Comparing the prevalence rate of PD at 41 per 100,000 among individuals aged 40–49 years, it was found that those aged 80 years and older exhibited a significantly higher prevalence rate of PD at 1903 per 100,000.^23^ As the resource from the Centers for Disease Control and Prevention (CDC) in the United States, the ALS prevalence rate was the lowest among individuals aged 18–39, with only 0.2 cases per 100,000 people. In contrast, the prevalence rate was highest in the 70–79 year age group, reaching 17.2 cases per 100,000 people.^24^ Multiple system atrophy (MSA) is a progressive neurodegenerative disorder that usually begins in the late 50 years to early 60 years. The prevalence of MSA increases with age, with a peak occurrence in individuals aged 50–70 years. Recent statistics indicate that MSA affects approximately 4.6 per 100,000 people aged 50–59 years, increasing to 7.8 per 100,000 in those aged 70–79 years.^25^ Corticobasal degeneration (CBD) typically manifests between the ages of 50 and 70 years. Its prevalence increases with age, with the most common onset occurring in the middle 60 years. Progressive supranuclear palsy (PSP) is another form of tauopathy commonly observed in individuals around their mid-60 years. Its prevalence notably increases with advancing age, often manifesting more prominently in those aged 70 years and older. This condition is characterized by the progressive accumulation of the tau protein in the brain, which becomes more prevalent with advancing age.^26^ FTLD is commonly identified in individuals between 45 and 65 years of age, yet its risk and occurrence increase with ageing. Research indicates an increased occurrence among older individuals, notably individuals aged 60 years and older.^27^ A retrospective analysis across Europe revealed that the average incidence of FTLD peaks at the age of 65–74 years, with 9.06 cases per 100000 person-years.^28^ Furthermore, according to an Italian epidemiological report, the prevalence rate of HD ranges from 4.35 per 100,000 individuals aged 40–44 years to 49.67 per 100,000 individuals aged 65–69 years.^29^

Given that ageing is a common risk factor for neurodegenerative diseases, a pivotal question arises regarding the mechanisms through which specific neurodegenerative diseases manifest in individuals as they age. Taking AD as an illustrative example, its onset during the ageing process is influenced by multiple factors. According to the widely accepted Aβ cascade hypothesis,^30^ neuronal Aβ is physiologically produced. However, an imbalance between Aβ production and clearance throughout the ageing process results in cerebral accumulation of Aβ, thereby facilitating the onset of AD. Furthermore, a confluence of genetic predispositions and environmental influences collectively shapes an individual’s unique trajectory towards AD progression, with ageing serving as a catalyst in this complex interplay. Alternative hypotheses for AD have also been proposed, including but not limited to the tau protein hypothesis,^31^ abnormal lipid^32^ and glucose metabolism hypothesis,^33^ inflammation hypothesis,^34^ oxidative stress hypothesis (mitochondrial dysfunction),^35^ and the cholinergic hypothesis.^36^ These frameworks suggest that the pathophysiology of AD is characterized by phosphorylated tau accumulation and propagation,^31^ heightened inflammatory responses, dysregulation of oxidative stress (related to mitochondrial dysfunction),^37^ alongside a gradual decline in cholinergic function.^38^ Notably, these events are intricately linked to the ageing process and synergistically contribute to the pathogenesis of AD.

**Fig. 1:**
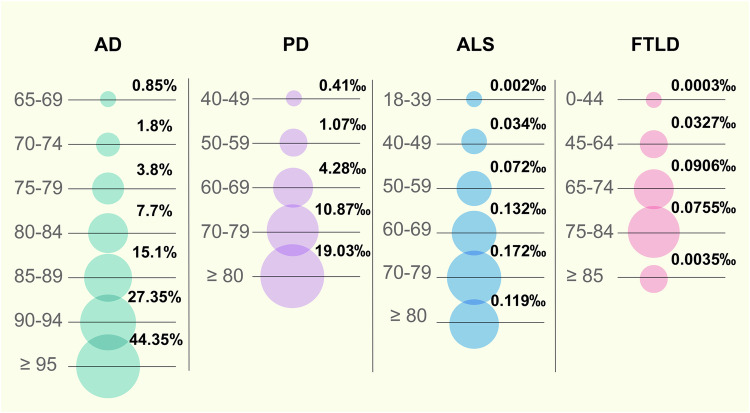
Prevalence or incidence of neurodegenerative diseases by age. Epidemiological evidence indicates that ageing is an accelerator of neurodegenerative diseases. The prevalence of AD was based on the data of 2000 in the United States and Europe. The global prevalence of PD was based on the data from 1985 to 2010. The prevalence of ALS was based on the data of 2016 in the United States. The incidence of FTLD was based on the data of 2021 in Europe. AD Alzheimer’s disease, PD Parkinson’s disease, ALS Amyotrophic lateral sclerosis, FTLD Frontotemporal lobar degeneration

Therefore, it can be inferred from the above epidemiological evidence that ageing is an accelerator of neurodegenerative diseases (Fig. 1). If we envision ageing as a flowing river, neurodegenerative disease is a boat navigating its waters. The flowing river increases the speed of the sailing boat. Comorbidities such as vascular diseases collide. Even if the oars stop moving, the boat continues to drift downstream as long as the river continues to flow. The analogy holds for the treatment of neurodegenerative diseases. Even if pathological proteins such as Aβ, hyperphosphorylated tau, α-syn and TDP-43 accumulation in the brain are effectively cleared, cognitive decline persists as the brain ages and comorbidities continue to interact. Consequently, solely targeting neurodegenerative disease-specific pathologic changes may not be sufficient to achieve the desired outcomes. Halting or reversing the flow of a river would be an effective approach to prevent the boat from moving forwards or even to facilitate it to move backwards. Similarly, a comprehensive approach that prioritizes systemic rejuvenation, alongside interventions targeting disease-specific pathogenic events, constitutes a promising disease-modifying strategy to “press the pause button” on dementia progression.

## Milestone events of studies on antiageing strategies

Significant breakthroughs have been made in the ageing and antiageing research fields; here, we review the research history and milestone events. As early as the 1930s, caloric restriction (CR) extended the lifespan of both mice and rats.^39^ Correspondingly, CR was found to prolong the healthy lifespan of rhesus monkeys in 2009.^40^ Since the mid-20th century, numerous ageing-related hypotheses and concepts have been proposed. In 1952, Peter Medawar et al. proposed the theory of ageing mutation accumulation, namely, that harmful mutations may continuously accumulate in an organism, eventually leading to ageing.^41^ In 1954, Denham Harman et al. proposed the free radical theory. In addition, he argued that reducing the production of free radicals could prolong the lifespan of mice by 20%.^42,43^ Then, George C. William et al. suggested the antagonistic pleiotropy theory that genes are favoured by natural selection if these genes exert beneficial effects on early fitness components as well as pleiotropic deleterious effects on late fitness components throughout life.^44^ Leonard Hayflick et al. discovered that the number of times a human cell can divide is limited, known as the “Hayflick limit” in 1961.^45^ Moreover, cellular senescence is defined as permanent growth arrest caused by endogenous and exogenous stress.^45^ Immunosenescence, a concept developed by Roy L. Walford et al. in 1969, is characterized by a decline in the body’s immune response to internal and external antigens.^46^ Later, in 1971, Alexey Olovnikov et al. pioneered the end-replication problem, which involves the loss of chromosome end fragments with each cell division, gradually shortening chromosomes.^47^ At the beginning of the 21st century, Claudio Franceschi et al. proposed the inflammageing theory.^48^ Moreover, the National Institute on Ageing (NIA) sponsored an intervention testing programme (ITP) to identify compounds that extend the lifespan of mice.^49^ A novel finding is emerging in this field: the awakening of endogenous retroviruses (ERVs) is a biomarker and powerful driver of cellular senescence and tissue ageing. In addition, targeting ERVs is a promising approach to alleviate ageing.^50^

In addition, many ageing-related genes and pathways have been identified. In 1988, AGE-1 mutation increased the lifespan of *Caenorhabditis elegans* by 40–60%.^51^ Similarly, the Daf-2 mutation doubled the lifespan of this species in 1993.^52^ Daf-2 inhibits insulin-like growth factor (IGF) intracellular signalling, which is involved in the regulation of blood glucose levels, suggesting that antiglucose drugs may interfere with ageing. In 2013, metformin prolonged the healthy lifespan of mice.^53^ The Food and Drug Administration (FDA) subsequently approved the clinical trial Targeting Ageing with Metformin (TAME). Moreover, in 1995, a sirtuin 4 (SIR4) mutation in yeast extended the lifespan by more than 30%,^54^ after which SIRT1 was verified in mammals.^55^ In 2003, small-molecule activators of sirtuins (SIRTs) extended the lifespan of yeast by 70%.^56^ Additionally, inhibition of the target of rapamycin (TOR) pathway prolonged the lifespan in a 2004 study.^57^ In 2009, rapamycin, an inhibitor of the mammalian target of rapamycin (mTOR) pathway, was shown to significantly extend the lifespan of mammals.^58^ In 2004, nicotinamide adenine dinucleotide (NAD^+^)-dependent sir2 was confirmed to extend the lifespan of Drosophila by 10–20%.^59^ Similarly, nicotinamide mononucleotide (NMN) is a direct precursor of NAD^+^, and the first clinical trial of NMN was conducted in 2021.^60^ A recent study demonstrated that metformin was capable of decelerating the ageing process of multiple organs in primates.^61^ In addition to these pathways, many transformations occur at the cellular level. In 1995, senescent cells were confirmed to exist and accumulate in human tissues with ageing, accompanied by senescence-associated secretory phenotypes (SASPs), which were proposed in 2008.^62^ In 2016, the elimination of senescent cells by senolytics in mice extended the healthy lifespan,^63^ and the first clinical trial was conducted in 2018.^64^ At the systemic level, the use of the young plasma to fight ageing was proposed early.^65^ In 2005, muscle regeneration and muscle stem cell viability in aged mice were restored by exposure to a young systemic environment.^66^

The concept of biological age emerged in the middle of the 20th century, specifically reflecting the degree of ageing of the structure and function of tissues/organs, and then, it was widely applied in the ageing research field.^67^ Supported by recent advances in high-throughput omics technologies, the first DNA methylation ageing clock was established in 2011 to assess biological age comprehensively and accurately.^68^ Later, the metabolomic ageing clock and transcriptomic ageing clock were published to explain ageing-related clinical traits.^69,70^ Steve Horvath et al. formally proposed the epigenetic clock in 2018. Genomic DNA methylation can be used to evaluate the methylation of a series of genetic loci and estimate the biological age.^71^

Antiageing strategies are increasingly being implemented in the context of AD and other neurodegenerative disorders. Preclinical studies have demonstrated that growth differentiation factor-11 (GDF11) in young plasma exerts neuroprotective effects by promoting neurogenesis within the hippocampus and enhancing learning and memory in aged mice.^72^ Two recent clinical trials involving young plasma infusion in patients with AD and PD, respectively, have further validated this intervention strategy for neurodegenerative diseases.^73,74^ Additionally, transplantation of young fecal microbiota has been shown to reverse age-related alterations in microglial activation while rejuvenating the metabolic profile of the hippocampus, primarily influencing amino acid metabolism. Furthermore, behavioural deficits were alleviated in older mice.^75^ Subsequent clinical trials have indicated that cognitive and behavioural improvements could be achieved through fecal microbiota transplantation (FMT) in patients with mild cognitive impairment as well as those suffering from PD.^76,77^ Moreover, clearance of senescent cells throughout the bodies of older mice led to a reduction in markers associated with neuronal senescence (such as LaminB1, P21, and High Mobility Group Box 1), reversal of age-related microglial activation and inflammation, enhancement of cognitive functions (including spontaneous activity and exploratory abilities), along with an extension of healthy lifespan among these older mice.^63,78^ The application of senolytics for patients diagnosed with early-stage AD has recently been investigated, yielding preliminary findings that suggest a potential role for these agents in managing neurodegenerative diseases.^79,80^

**Fig. 2:**
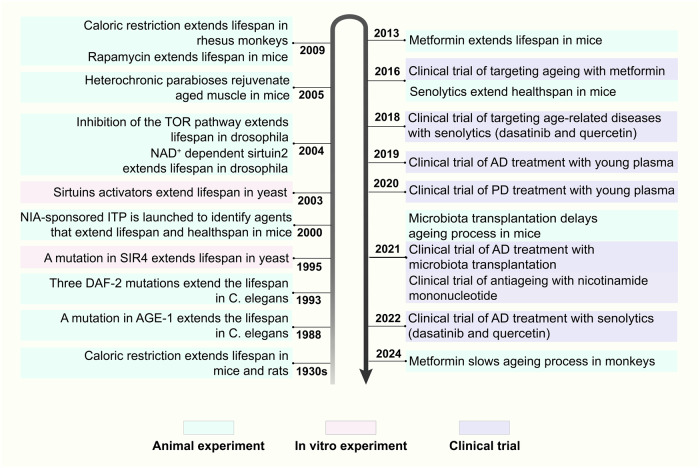
Milestone events in the history of antiageing research. This figure enumerates key events in the field of antiageing research and pivotal advancements in unitizing antiageing strategies to intervene neurodegenerative diseases from the 1930s onwards. NIA National Institute on Aging, ITP Intervention Testing Program, SIR4 sirtuin 4, NAD+ nicotinamide adenine dinucleotide, TOR target of rapamycin, C. elegans Caenorhabditis elegans, AD Alzheimer’s disease, PD Parkinson’s disease

Physiological ageing and neurodegenerative diseases is inevitable and will continue to drive persistent research. In recent years, due to advances in high-throughput single-cell omics technologies and large-scale profiling,^81^ the research paradigm has shifted. The molecular features and mechanisms of ageing and neurodegenerative diseases have been analysed in unprecedented depth and comprehensiveness. These findings lay the foundation for subsequent studies on precise humoral markers of ageing and neurodegenerative diseases and effective targets for prevention and treatment (Fig. 2).

## Complex systems view of ageing and neurodegenerative diseases

Ageing, neurodegenerative diseases and their comorbidities are characterized by a multifactorial and complex nature. According to the concepts of complex systems science, the human body is a self-organizing complex adaptive system (CAS), namely, a “network of networks”.^82^ These networks include horizontal connections among molecules, cells, organs, systems, and individuals, as well as vertical connections within each layer. Through the dynamic regulation of this high-dimensional and multiscale network, the human body actively and adaptively responds to internal or external stimuli to maintain homoeostasis, function and health.^83,84^ During ageing, adaptive responses are weakened due to deficits in the CAS network.^83^ When a stimulus is too intense and exceeds the regulatory capacity of the adaptive response or when the compromised adaptive response is insufficient to recover from stimulus-induced perturbations, homoeostasis is disrupted, leading to the onset of disease.^83–85^ The intricate nature of ageing substantiates that ageing constitutes a nonlinear dynamic process characterized by variability across different organs and systems.^86^

**Fig. 3:**
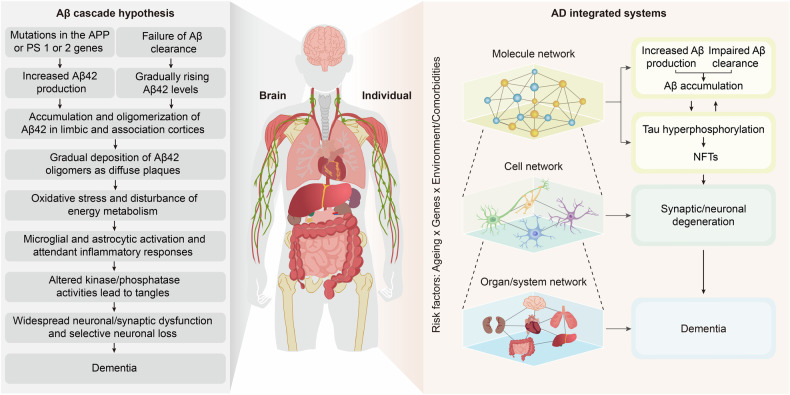
Overview of integrated systems involved in AD pathogenesis. AD was initially proposed to obey the Aβ cascade hypothesis, with Aβ as its core. Dysregulation of Aβ production and clearance leads to its accumulation, which further induces downstream oxidative stress, NFT formation, neuronal and synaptic degeneration, and ultimately dementia. Here, we propose viewing AD from the perspective of integrated systems. When intense stimulation exceeds the body’s resistance or when the resilience is insufficient to recover from the stimulus-induced disruption, homoeostatic imbalances result, as evidenced by Aβ and tau accumulation, synaptic/neuronal degeneration, and dementia. Genes, the environment and lifestyle are involved at every level. AD Alzheimer’s disease, APP amyloid precursor protein, Aβ amyloid-β, PS presenilin, NFTs neurofibrillary tangles. The figure was produced utilizing the applications Easy PaintTool SAI and Adobe Illustrator

The concept of complex systems science is not foreign to neurodegenerative diseases. Take AD as an example (Fig. 3). The prevailing Aβ cascade hypothesis for AD suggests that an imbalance in the production and clearance of Aβ leads to its deposition. Aβ deposition initiates a series of downstream pathological events, such as tau pathology, oxidative stress, and energy metabolism disorders, ultimately resulting in synaptic or neuronal degeneration and eventually dementia.^30,87^ This linear hypothesis elucidates the major pathologic outcomes that arise from imbalances in homoeostasis at various scales, from the molecular to the cellular and ultimately to the organ layers, as well as the interconnections among them. In fact, the homoeostatic imbalances at each scale are a result of the dysregulated CAS. For example, Aβ deposition is the result of a homoeostatic imbalance between a continuous and intense stimulus (stressors leading to the overproduction or impaired clearance of Aβ) and an inadequate adaptive response (suppression of responses to inhibit the production or enhance the clearance of Aβ), as discussed below. Tau pathology is the result of an imbalance between tau phosphorylation and dephosphorylation induced by various triggers,^88^ principally Aβ. Neuronal degeneration involves increased amounts of neurotoxic molecules (e.g., Aβ, hyperphosphorylated tau, and inflammatory factors) and an insufficient supply of energy and neurotrophic factors,^89,90^ which are derived from various neural cells and tissues or organs other than the brain, as well as defective resistance or resilience of neurons to stressors.^91^

Similarly, deficits in the CAS network also contribute to PD. PD is pathologically marked by intracellular aggregates of α-syn, known as Lewy bodies, and is characterized by the loss of dopaminergic neurons in the substantia nigra pars compacta. Like Aβ deposition in AD, the abnormal aggregation of α-syn results from its overproduction (due to gene mutations, abnormal posttranslational modifications, and elevated oxidative stress) and the failure of α-syn clearance (due to proteasome and autophagy dysfunction). The degeneration of dopaminergic neurons is caused primarily by a combination of impaired proteostasis and mitochondrial dysfunction.^92^ These molecular and cellular stresses converge to trigger apoptotic and other cell death pathways, ultimately resulting in the progressive loss of dopaminergic neurons and the manifestation of PD.

In addition, a defective CAS network is common in other neurodegenerative diseases. ALS, known as idiopathic fatal motor neuron disease, is characterized by the degeneration of both upper and lower motor neurons, leading to progressive muscle weakness, atrophy and eventual paralysis.^93^ FTLD, one of the most common types of dementia, is characterized by the degeneration and atrophy of the frontal and temporal lobes of the brain and presents with early social–emotional–behavioural and/or language changes, accompanied by pyramidal or extrapyramidal motor neuron dysfunction.^94^ Although ALS and FTLD have different disease manifestations, many genetic and pathological mechanisms overlap. One specific pathology is the accumulation of TDP-43 within affected neurons. The aggregation of TDP-43 is due to its overproduction (due to SOD1, TDP-43 and FUS mutations) and the impaired cellular homoeostasis of motor neurons (e.g., mitochondrial function, axonal transport and RNA metabolism). Moreover, glial cells are actively involved in ALS/FTLD pathology in a noncell autonomous manner, affecting TDP-43 accumulation and subsequent neurodegeneration. For example, low levels of phagocytosis and autophagy, as well as the secretion of inflammatory factors by microglia, are associated with the noncell-autonomous toxicity of astrocytes.^95^ Furthermore, HD is a progressive neurodegenerative disorder with a distinct phenotype, including chorea and dystonia, incoordination, cognitive decline, and behavioural difficulties.^96^ The pathogenesis of HD primarily stems from an abnormally expanded CAG repeat near the N-terminus of the Huntingtin gene, resulting in the production of mutant HTT (mHTT) protein. This aberrant protein forms toxic aggregates, which further disrupts the protein degradation system, axonal transport, and mitochondrial function. Additionally, glial cell activation synergistically contributes to neurodegeneration.^97,98^

Overall, the development of neurodegenerative disorders does not result from a single molecule or cell but rather from an emergent homoeostatic imbalance within CAS. The integrated systems perspective provides a comprehensive approach to understanding the pathogenesis of neurodegenerative disorders. Understanding these interconnected processes is essential for developing effective strategies to prevent and treat age-related neurodegenerative diseases.

## Systemic regulatory mechanisms of ageing in neurodegenerative diseases

In this section, we discuss the impacts of ageing on neurodegenerative diseases from the perspective of complex systems.

### Molecular and cellular networks of ageing in neurodegenerative diseases

#### Changes in the ageing brain

Brain ageing involves multidimensional and multilevel changes in molecules, cells, neural circuits, tissues, and brain functions. The hallmarks of ageing have been newly refined and include genomic instability, telomere attrition, epigenetic alterations, loss of proteostasis, disabled macroautophagy, deregulated nutrient sensing, mitochondrial dysfunction, cellular senescence, stem cell exhaustion, altered intercellular communication, chronic inflammation and dysbiosis.^85^ The hallmarks of brain ageing are broadly consistent with these hallmarks, but specific characteristics have been identified, such as aberrant neural network activity and glial cell activation.^99^ This section summarizes the hallmarks of brain ageing based on these aspects.

##### Molecular level

Brain ageing involves several critical molecular changes that collectively contribute to cognitive decline and increased susceptibility to neurodegenerative diseases. One of the hallmarks of brain ageing is the elevated levels of oxidative stress.^100,101^ The production of reactive oxygen species (ROS) increases, leading to oxidative damage to cellular components such as DNA, proteins, and lipids. The efficiency of DNA repair mechanisms decreases with age, leading to the persistence of DNA lesions.^102^ These impairments can disrupt gene expression and cellular function. Changes in DNA methylation, histone modification, and noncoding RNA expression have also been observed and affect gene expression profiles in ageing neurons.^103,104^ Chronic sterile low-grade inflammation, often referred to as “inflammageing” is another significant molecular feature. Glial cells in the brain, such as microglia and astrocytes, become chronically activated and release proinflammatory cytokines.^105^ Mitochondrial function deteriorates with age, resulting in decreased ATP production and increased ROS generation.^106^ Mitochondrial DNA mutations accumulate, further impairing cellular energy metabolism and promoting apoptotic pathways.^107^ Additionally, the process of brain ageing is accompanied by the accumulation of misfolded proteins associated with neurodegenerative diseases, such as deposition of Aβ and α-syn, accumulation of hyperphosphorylation of tau (i.e. primarily ageing-related tauopathy, PART),^108^ and pathology of TDP-43 (i.e. limbic predominant age-related TDP-43 encephalopathy, LATE).^109^

The nutrient-sensing network is highly conserved throughout evolution and is deregulated during ageing. The insulin/insulin-like growth factor 1 (IGF-1) signalling (IIS) pathway is the first identified and extensively validated age-regulating pathway.^110^ IGF-1 is expressed in neurons and glial cells in a brain region-specific manner and has a neuroprotective effect by promoting synaptogenesis and neurotrophin signalling,^111^ counteracting oxidative stress and inflammation, and modulating neuronal excitability.^112^ However, during ageing, a decrease in the activity of IGF-1 occurs, which manifests as deficiency and resistance, and exacerbates age-related changes in the brain.^113–115^ In addition, mTOR, known as a modulator of key cellular processes, participates in the activation of protein synthesis, biomass accumulation and the repression of autophagy.^116^ The activity of mTOR in the brain increases with age,^117^ substantially inhibiting autophagy, which could explain why pathological proteins are prone to accumulation.^118^ Furthermore, seven mammalian SIRTs, namely, sirtuin1 to sirtuin7, have been identified. SIRTs are involved in various biological processes, including inflammation, glucose and lipid metabolism, oxidative stress, cell apoptosis, and autophagy.^119^ SIRT1, one of the most valuable targets, deacetylates protein substrates to exert neuroprotective effects, maintaining neural integrity.^120^ SIRT1 transcription decreases in the aged brain,^121^ worsening pathological protein aggregation and neuron loss and sharply increasing the risk of neurodegenerative diseases.^122,123^ Similarly, alterations in other ageing-related pathways, such as adenosine 5’-monophosphate-activated protein kinase (AMPK) and NAD^+^, with ageing also contribute to neurodegeneration.^124,125^ Notably, these pathways do not act singly but rather interact with each other to regulate ageing and ageing-related diseases. For example, SIRT1 downregulates the mTOR pathway and upregulates the AMPK pathway, synergistically enhancing autophagy.^126^

##### Cellular level

The accumulation of molecular changes leads to structural and functional alterations in various brain cells. Neuronal dendrites, which receive synaptic inputs, can retract and lose their complexity, reducing the number of synaptic connections.^127^ A reduction in synaptic density has been observed, which impacts neural communication. Ageing-related pigments, such as lipofuscin, accumulate within neurons. Functionally, the synthesis and release of neurotransmitters (e.g., acetylcholine, dopamine, and glutamate) and neurotrophic factors (e.g., NGF and BDNF) decrease.^128^ Neuronal excitability and plasticity decline, and metabolic activity, such as ATP production, is reduced.^129^ Additionally, senescent cells and SASPs accumulate in the ageing brain to drive neurodegeneration.^130^

Brain ageing also involves functional and structural changes in various glial cells. The process of brain ageing is characterized by inflammation, with microglia serving as crucial immune regulatory cells in the brain, indicating their significant role in this process. The states of microglia, such as telomerase activity,^131^ morphology and distribution pattern,^132^ degree of activation,^133^ cell migration and the speed of the response to inflammation,^134^ significantly change with ageing. During the ageing process, there is a significant increase in neurotoxic M1 microglia, accompanied by a concomitant decrease in neuroprotective M2 microglia. This imbalance leads to the production of substantial quantities of pro-inflammatory factors, chemokines, and reactive substances, collectively exacerbating neuroinflammation.^135^ The initial identification of disease-associated microglia (DAM) occurred in the brain tissue of AD transgenic mice,^136^ with subsequent research indicating that the prevalence of DAM cells increases with age. High-dimensional cytometry revealed that approximately 11.9% of microglia in aged mice were classified as DAM, while no DAM cells were detected in young mice.^137^ High-throughput sequencing revealed that the expression of genes related to cell migration and cytoskeletal protein homoeostasis in aged microglia changed significantly,^138^ explaining the decrease in microglial migration caused by ageing. Moreover, microglia are the main cells responsible for the clearance of pathological substances and cell debris in the brain, but this clearance capacity decreases significantly with ageing.^139^

Astrocytes play an indispensable role in maintaining the homoeostasis of the nervous system. Changes in the gene expression and structure of astrocytes are early events in brain ageing.^140,141^ During the ageing process, astrocytes exhibit ageing-related phenotypes, such as an increased stress response, reduced telomere length and mitochondrial activity,^142^ and their ability to maintain neuronal activity and promote the proliferation of neural precursor cells is also significantly reduced.^143,144^ Under inflammatory conditions, astrocytes may be transformed into the neurotoxic A1 state or the neuroprotective A2 state.^145^ During ageing, astrocytes spontaneously transform into a neurotoxic A1 state,^146^ which in turn causes neuronal dysfunction. The regulatory mechanism underlying this transformation needs to be further explored. As astrocytes regulate homoeostasis in the central nervous system (CNS), changes in the astrocyte states may directly impair neuronal function and ultimately lead to the occurrence of various neurodegenerative diseases.

Oligodendrocytes are located in the white matter of the brain and protect the integrity of axons by forming myelin structures on the surface of neuronal axons. Consistent with astrocytes, as the brain ages, changes in gene expression in oligodendrocytes precede those in neurons and microglia,^140^ and their dysfunction may increase the vulnerability of neurons to ageing-related pathogenic risk factors. Ageing is often accompanied by a demyelination process, which is associated with increased levels of DNA oxidative damage in aged oligodendrocytes.^147^ The myelinogenesis and remyelination capacities of oligodendrocyte precursor cells (OPCs) also decrease.^148^

The blood‒brain barrier (BBB) is a critical structure that protects the brain by regulating the entry of substances from the bloodstream into neural tissue. The BBB also functions as part of the neurovascular unit (NVU), which is composed of astrocytes, microglia, specialized endothelial cells, pericytes, and the basement membrane of the BBB. The endothelial cells that line the blood vessels in the brain become less effective with age. An increase in the permeability of these cells can lead to a compromised barrier.^149^ The basement membrane, which supports endothelial cells, also undergoes thickening and structural alterations.^150^ These changes compromise the structural integrity of the barrier. The expression and functionality of proteins that form tight junctions between endothelial cells, such as occludin and claudin, are reduced.^151,152^ The structure and function of the BBB also undergo significant alterations with ageing, which contribute to the progression of neurodegenerative diseases and cognitive decline.^153^ A key change is the increased permeability of the BBB.^154^ The enhanced permeability facilitates the easier entry of potentially detrimental substances, such as toxins and pathogens, into the brain. The process of ageing is associated with a state of low-grade chronic inflammation, which further compromise the integrity of BBB, leading to increased permeability and more significant damage. The glymphatic system, a crucial transportation mechanism, facilitates the clearance of metabolic waste and misfolded proteins within the brain. This system is comprised of three distinct components: the periarterial space, the perivenous space, and the interstitial space in brain parenchyma. The expression of aquaporin-4 (AQP4) in astrocytes significantly influences the transport and clearance functions of the glymphatic system.^155^ As individuals age, there is a decline in the efficiency of these transport mechanisms in eliminating waste products such as Aβ.^156,157^ This inefficiency contributes to the accumulation of neurotoxic substances within the brain during ageing.

Notably, changes in various brain cells during ageing are not independent events. In contrast, these cells closely interact with each other through cell‒cell cross talk and jointly promote brain ageing and brain ageing-related neurodegeneration. For example, demyelination is an early sign of brain ageing, and shed myelin sheaths can accumulate in microglia, leading to microglial ageing and dysfunction.^158^ Senescent microglia actively secrete proinflammatory cytokines, which further leads to activation of astrocyte and neuronal apoptosis.^145^ The current understanding of the ageing hallmarks in various brain cells is comprehensive; therefore, exploring the characteristics of altered intercellular communication during ageing should be considered as a prospective avenue for future research.

##### Tissue or organ level

The accumulation of cellular changes gradually leads to alterations in brain structure. Changes in brain volume and structure are significant characteristics of brain ageing. One of the most notable structural changes in the ageing brain is thinning of the cerebral cortex.^159^ Studies using magnetic resonance imaging (MRI) have consistently shown a reduction in cortical thickness with age.^160^ This thinning is particularly evident in the prefrontal cortex, which is responsible for executive functions such as decision-making, problem-solving, and planning.^161^ Ageing is associated with a decrease in grey matter volume, which consists of neuronal cell bodies, dendrites, and synapses. The reduction in the grey matter volume is more pronounced in regions such as the hippocampus, which plays a critical role in memory and learning.^162^ The white matter, which contains myelinated axons, also undergoes significant changes. The white matter integrity undergoes a general decline, characterized by decreases in both myelin density and quality.^163^ This degradation can lead to slower neural signal transmission and impaired connectivity between different brain regions.

##### Neuronal circuit level

Functional connectivity between different brain regions changes with age. A decrease in the strength of long-range connections, particularly between the frontal and parietal lobes, often occurs.^164^ Imaging studies have revealed a decrease in the fraction of action-plan-coding neurons and the action plan signal of individual neurons in the medial prefrontal cortex (mPFC), leading to impaired working memory coding and recurrent connectivity.^165^ Conversely, an increase in local connectivity may be present within certain regions, which can lead to less efficient information processing. The cognitive function arises from the dynamic interactions occuring within extensive brain networks. Studies have shown that intranetwork connectivity decreases while extranetwork connectivity increases with age, diminishing the integrity of many large-scale networks.^166^ The default mode network (DMN), which is active during rest and is involved in self-referential thinking, shows altered activity patterns with ageing. Older adults often exhibit decreased deactivation of the DMN during task performance, which is thought to contribute to attentional deficits.^167^ The circuitry of the hippocampus, crucial for memory formation, undergoes alterations. A decrease in the functional connectivity between the hippocampus and other brain regions, such as the prefrontal cortex, has been observed.^168^ These disruptions can impair the encoding and retrieval of memories. Disruptions in primary information processing networks, such as auditory, visual, and sensorimotor networks, may lead to the overactivity of multisensory integration networks and the accumulation of pathological proteins, contributing to the development of dementia.^169^ Ageing also affects various neurotransmitter systems, including those involving dopamine, serotonin, and acetylcholine. The activity of dopaminergic circuits, specifically, exhibits a decline, thereby potentially impacting motor control and executive functions.^170^

##### Functional level

The cumulative alterations occurring at the aforementioned levels during the process of ageing ultivately result in impairments in brain function, which serve as the basis of various neurodegenerative diseases. Brain functions, including cognition, motor coordination, sensory perception, and emotion, are affected by ageing. Ageing is strongly associated with a decline in cognitive functions, including memory, executive function, processing speed, and attention. Episodic memory and working memory are particularly susceptible to age-related decline, which adversely affects an individual’s capacity for acquiring new information and excuting intricate cognitive tasks.^171^ The decline in fine motor ability is consistently observed with advancing age,^172^ making it a reliable indicator for predicting brain ageing. Additionally, emotional changes, such as age-related anxiety and depression, are prevalent in ageing populations.^173,174^

In conclusion, the hallmarks of brain ageing involve multiple factors, ultimately leading to a decline in overall nervous system function. These hallmarks provide a crucial basis for assessing the degree of brain ageing and for the prevention and treatment of neurodegenerative diseases.^175^

#### Regulatory mechanisms of brain ageing in neurodegenerative diseases

**Fig. 4:**
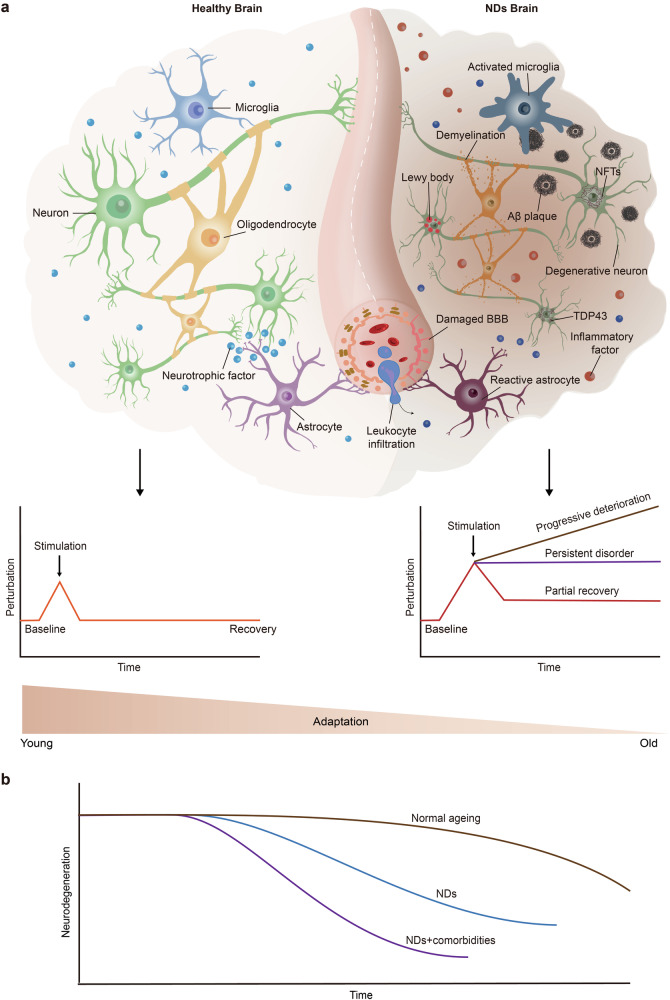
Schematic diagram of the decline in brain adaptation during ageing and neurodegenerative diseases. **a** Young and healthy brains can actively respond to various stimuli, thus maintaining homoeostasis and normal brain function. During ageing, the compromised adaptation of the brain is insufficient to recover from stimulus-induced perturbations, resulting in a homoeostasis imbalance and the development of disease. **b** In the brains of neurodegenerative disease patients, pure neurodegenerative disease pathology is relatively rare and is often accompanied by other pathological changes, such as vascular damage and the aggregation of pathological proteins (e.g., TDP43 and Lewy bodies). Once these comorbidities occur, cognitive decline appears earlier, progresses more rapidly, and reaches lower levels. NDs neurodegenerative diseases, Aβ amyloid-β, BBB blood–brain barrier, NFTs neurofibrillary tangles, TDP43 transactive response DNA binding protein 43. The figure was produced utilizing the applications Easy PaintTool SAI and Adobe Illustrator

Brain ageing is the principal risk factor for a spectrum of neurodegenerative diseases. It precipitates the onset of these conditions through a convergence of cellular and molecular processes, notably oxidative stress, inflammation, disrupted proteostasis, synaptic dysfunction, compromise BBB integrity, genetic predisposition, and cellular senescence. Although ageing constitutes a unifying factor in neurodegeneration, each disorder manifests distinct pathological and molecular signatures. AD ranks among the most prevalent neurodegenerative conditions. Accordingly, our focus lies in elucidating the molecular and cellular mechanisms through which brain ageing influences AD. Additionally, we delineate ageing-associated mechanisms pertinent to other neurodegenerative disorders, such as PD, ALS, and HD (Fig. 4).

##### Brain ageing and AD

Brain ageing and AD share common alterations, such as a loss of proteostasis, oxidative stress, and inflammation, which are exacerbated in AD.^99^ These alterations are caused by cellular dysfunction and involve almost all types of neural cells. The neurons serve as the principal site for Aβ production and NFT formation, which are fundamental to cognitive impairment. The amyloidogenic processing of APP primarily involves the activities of β- and γ-secretases, both of which are upregulated in neurons during ageing.^176^ Conversely, the activity of Aβ-degrading enzymes such as neprilysin and insulin-degrading enzymes decreases,^177^ thereby promoting cerebral Aβ accumulation. Microglia are the primary immune cells in the brain that clear pathological and redundant substances such as Aβ. However, the phagocytosis of Aβ is defective in aged microglia.^178^ Astrocytes play a crucial role in providing neurons with energy and neurotrophic factors, while also being involved in the regulation of the BBB funciton and inflammatory processes.^179^ The process of ageing in astrocytes results in neuronal energy and nutrient deficiency, an augmented SASP and BBB permeability.^90^ Furthermore, oligodendrocytes provide energy and nutrients for neuronal axons and protect them from injury. The loss of myelin integrity with ageing has been reported to promote Aβ formation and neuronal degeneration in animal models.^180^ Cerebral vessels, especially microvessels are responsible for the exchange of substances between the brain and the blood. The integrity and functionality of the BBB and the lymphatic system are contingent upon the structural characteristics and operational dynamics of cerebral blood vessels.^149,155^ The permeability of the BBB increases and the transport capacity of the glymphatic system diminishes during the process of age, facilitating the accumulation of Aβ and other pathological substances in the brain.^181,182^

Importantly, brain cells do not function independently but interact in the form of cellular networks such as neurovascular units. During the process of brain ageing, the concurrent decline in both the structure and function of these neural cells, along with the presence of comorbidities, results in an imbalance between the stimulation and adaptive responses of the CAS, leading to accumulation of Aβ and tau proteins, neurodegeneration, and ultimately dementia. Thus, the role of age-related alterations in intercellular communication in the AD pathogenesis is worthy of investigation.

Furthermore, instances of concurrent pathologic changes are prevalent in elderly individuals, whereas pure AD represents an exception. The most common comorbidities that underlie cognitive impairment include pathologic changes associated with cerebrovascular and other concomitant neurodegenerative diseases (e.g., Lewy bodies, TDP-43, and hippocampal sclerosis). Data from the Religious Orders Study/Memory and Ageing Project (ROS/MAP) cohort revealed that approximately 97% of persons diagnosed with probable AD had other concomitant neurodegenerative or vascular comorbidities, including microinfarcts or any of the vessel diseases that are also commonly present and contribute to cognitive impairment, whereas more than 86% of older persons without cognitive impairment had vascular, AD or other degenerative comorbidities in the brain.^183^ These comorbidities are also affected by ageing and promote the progression of AD and dementia.

##### Brain ageing and PD

Several major molecular hallmarks of brain ageing overlap with mechanisms implicated in PD neurodegeneration, including oxidative damage and mitochondrial dysfunction, a loss of protein homoeostasis, neuroinflammation, genomic instability, and impaired stress responses. Among them, mitochondrial dysfunction and bioenergetic failure have been implicated as primary mechanisms for the development of PD. This finding is supported by the identification of reduced levels of complex I in dopaminergic neurons of PD patients,^184^ and reinforced by recent studies on familial PD-linked genes such as,leucine-rich repeat kinase 2 (LRRK2), Parkin, synuclein alpha (SNCA), and DJ-1, as well as PD-like phenotypes resulting from genetic deletion of a catalytic ETC complex I subunit.^184–187^ Dopaminergic neurons are more vulnerable to the age-related loss of mitochondrial function, resulting in bioenergetic stress due to their highly ramified processes that harbour dense mitochondria to sustain energy-requiring processes at distal sites.^188^ Ageing is a critical factor contributing to mitochondrial dysfunction, a pivotal event in the pathogenesis of PD. As cells age, mitochondrial DNA accumulates mutations, and the efficiency of oxidative phosphorylation decreases.^189^ These changes lead to increased production of ROS, causing oxidative stress and damage to cellular components, including proteins, lipids, and nucleic acids. Furthermore, ageing impairs the mitophagy process, reducing the clearance of damaged mitochondria and exacerbating cellular stress.^190^ These vulnerabilities are particularly pronounced in the dopaminergic neurons of the substantia nigra due to their high metabolic demand and reliance on mitochondrial function. The convergence of these ageing-related mitochondrial impairments contributes significantly to the neurodegenerative processes observed in PD patients, highlighting the importance of maintaining mitochondrial health as a potential therapeutic avenue for mitigating disease progression.

##### Brain ageing and other neurodegenerative diseases

In addition to AD and PD, ALS, FTLD and HD are also prominent neurodegenerative disorders. This section provides a concise overview of the ageing mechanisms implicated in ALS, FTLD, and HD, with a specific focus on how the process of ageing influences their distinct pathological progression.

Unlike AD and PD, ALS is a relatively rare neurodegenerative disease with a global prevalence of approximately 1.57–11.80 per 100,000 individuals.^191^ The average age of onset is 55 years. Ageing intersects with unique molecular mechanisms in ALS that differentiate it from other neurodegenerative conditions. One distinguishing characteristic is the preferential susceptibility of motor neurons to protein aggregation. Mutations in genes such as SOD1, TDP-43, and FUS lead to the formation of toxic protein aggregates specifically within motor neurons.^192^ The cellular capacity for efficient trafficking and clearance of misfolded proteins diminishes with age, resulting in the accumulation of toxic proteins and hastening the demise of motor neurons. Furthermore, ALS is characterized by aberrant RNA processing and nucleocytoplasmic transport defects, which are often linked to mutations in C9orf72 and other RNA-binding proteins.^193^ Ageing-related changes in the expression and activity of splicing factors can further impair RNA processing. Unlike other neurodegenerative diseases, ALS also results in pronounced disturbances in the axonal transport and cytoskeletal dynamics of motor neurons.^194^ These molecular abnormalities, coupled with age-related decreases in cellular repair mechanisms, result in the progressive degeneration of motor neurons, underscoring the unique interplay between ageing and ALS pathogenesis.

FTLD, which shares some of the pathological and genetic mechanisms with ALS, is also a common form of dementia, with a prevalence ranging from 1 to 461 per 100,000 people.^195^ The majority of FTLD cases arise from mutations in genes encoding microtubule-associated protein tau (MAPT), progranulin (GRN), and C9orf72, whereas the remaining FTLD cases are caused primarily by mutations in genes encoding FUS, TDP-43, valosin-containing protein (VCP) and charged multivesicular body protein 2B (CHMP2B). These mutations are associated with defective autophagic clearance and lysosomal function.^196^ Autopsy evidence revealed that the brains of the elderly population are more susceptible to TDP43 accumulation.^197^ Additionally, brain ageing is accompanied by lysosomal dysfunction and neuroinflammation,^198^ which collectively accelerate the occurrence and development of FTLD.

HD is a relatively rare neurodegenerative disease, with an average prevalence of 4.88 per 100,000 individuals.^199^ HTT gene mutations trigger a cascade of molecular events, including transcriptional dysregulation, impaired protein homoeostasis, and disrupted intracellular transport.^200^ These abnormalities are compounded by age-related decreases in cellular repair mechanisms and increased oxidative stress. Unlike other neurodegenerative diseases, HD specifically affects the striatum and cortex, leading to characteristic motor, cognitive, and psychiatric symptoms. Thus, the interplay between ageing and the unique genetic and molecular landscape of HD drives its distinct pathogenesis.

**Fig. 5:**
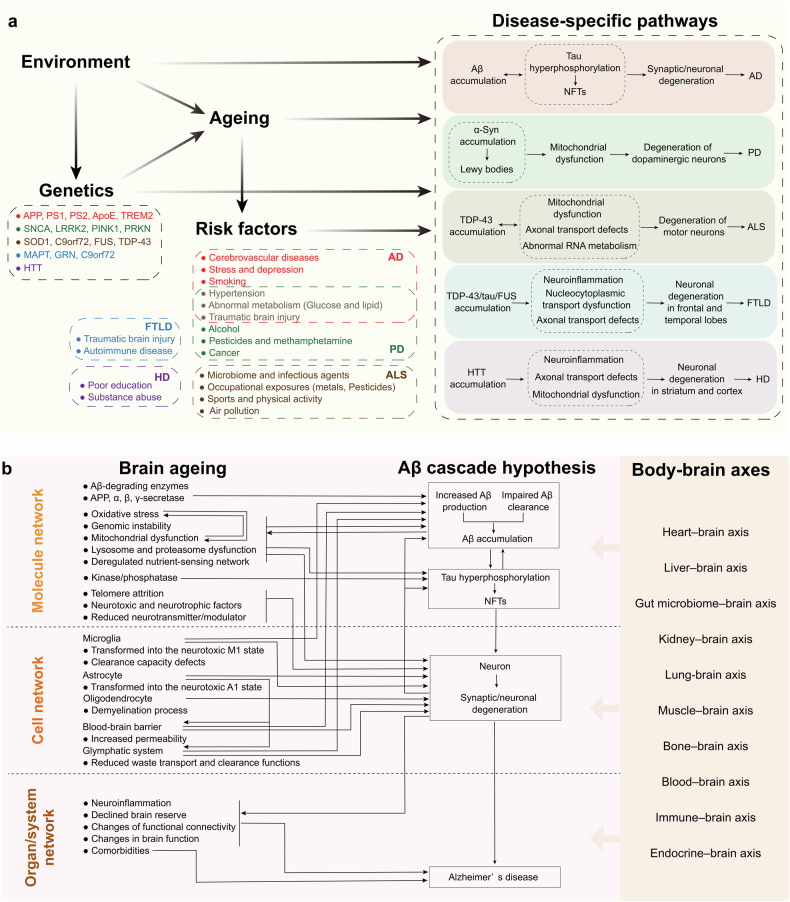
Specific mechanisms by which ageing promotes different neurodegenerative diseases. **a** Ageing promotes specific neurodegenerative diseases. Ageing is a holistic non-specific process that nevertheless facilitates the emergence of distinct types of neurodegenerative diseases in different individuals. Ageing is co-regulated by both environment and genetic factors (wherein the latter are also subject to environment). As well, ageing exerts an effect on the specific risk factors associated with different neurodegenerative diseases. The interplay between environmental and genetic factors co-regulates ageing, with the latter also being influenced by environmental conditions. Furthermore, ageing impacts the specific risk factors associated with different neurodegenerative diseases. These four aforementioned aspects synergistically interact with the unique mechanisms and pathways underlying neurodegenerative diseases. **b** Brain ageing acts on the AD pathway. During brain ageing, molecular, cellular, and tissue/systemic networks undergo profound transformations that promote specific pathways conducive to various neurodegenerative diseases. For example, AD is characterized by Aβ accumulation, which occurs alongside age-related comorbidities leading to neuronal degeneration and AD progression. In the context of brain ageing, Aβ-degrading enzymes and amyloidogenic processing of APP directly affect both Aβ production and clearance rates. Key hallmarks of ageing include oxidative stress, mitochondrial dysfunctions, genomic instability, proteasome and lysosomal dysfunctions as well as nutrient perception disorders; these factors collectively enhance Aβ deposition. Additionally, age-related reductions in microglial activity and transport systems such as the blood-brain barrier and glymphatic system impair Aβ clearance efficiency. The accumulation of Aβ triggers downstream formation of NFTs, further exacerbating hallmark features associated with ageing while concurrently diminishing neuroglial support for neurons, this combination accelerates neuronal degeneration linked to AD pathology. Moreover, neuroinflammation along with alterations in structural integrity and functional capabilities within an ageing brain contribute significantly to AD pathogenesis; peripheral organ ageing also plays a role in influencing AD progression through direct effects on Aβ dynamics as well as indirect effects on brain ageing. APP amyloid precursor protein, PS presenilin, ApoE Apolipoprotein E, TREM2 triggering receptor expressed on myeloid cells 2, SNCA synuclein alpha, LRRK2 leucine-rich repeat kinase 2, PINK1 PTEN-induced putative kinase 1, PRKN parkin RBR E3 ubiquitin protein Ligase, SOD1 superoxide dismutase 1, C9orf72 chromosome 9 open reading frame 72, FUS fused in sarcoma, TDP-43 transactive response DNA binding protein 43, MAPT microtubule associated protein tau, GRN granulin, HTT huntingtin, AD Alzheimer’s disease, PD Parkinson’s disease, ALS Amyotrophic lateral sclerosis, FTLD Frontotemporal lobar degeneration, HD Huntington’s disease, NFTs neurofibrillary tangles, Aβ amyloid-β, α*-s*yn α-synuclein

Ageing is a holistic non-specific process that nevertheless promotes specific types of neurodegenerative diseases in different individuals. Ageing is regulated by both environment and genetic factors (wherein the latter are also subject to environment). As well, ageing exerts an effect on the specific risk factors associated with different neurodegenerative diseases. The four aforementioned aspects act in a synergistic manner on the specific mechanisms and pathways of neurodegenerative diseases. The molecular, cellular, and systemic regulatory mechanisms of brain ageing significantly contribute to the development and progression of various neurodegenerative diseases. Key mechanisms encompass genetic factors, neuroinflammation, oxidative stress, mitochondrial dysfunction, proteostasis disruption, protein aggregation, synaptic plasticity impairment, and cellular senescence. Collectively, these mechanisms modulate specific pathways involved in neurodegenerative diseases. In the context of AD, brain ageing processes play a crucial role in neuronal degeneration and disease progression through their pleiotropic impact on AD-specific pathologies (such as amyloid-beta accumulation and tau hyperphosphorylation) as well as common age-related changes (Fig. 5). Understanding these intricate mechanisms provides essential insights into potential therapeutic strategies aimed at mitigating the effects of ageing on the brain and slowing down the progression of neurodegenerative disorders.

### Body‒brain axes in relation to ageing and neurodegenerative diseases

**Fig. 6:**
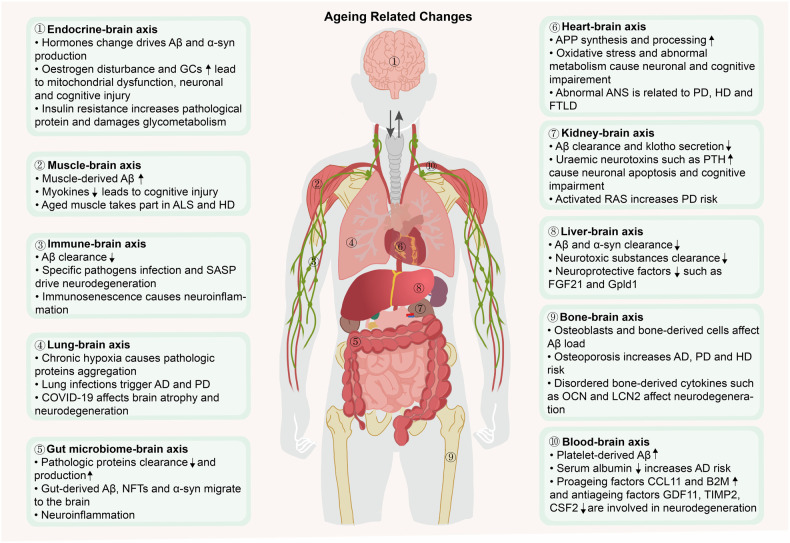
The impacts of the body‒brain axis ageing on neurodegenerative diseases. The brain interacts with multiple peripheral organs, and the functions and structures of peripheral organs change with age, leading to a decline in their support of the brain. Aged peripheral organs interfere with pathological proteins accumulation, neuronal activity and other brain functions, ultimately promoting the dysregulation of brain homoeostasis and the occurrence of neurodegenerative diseases. FSH follicle-stimulating hormone, Aβ amyloid-β, GCs glucocorticoids, SASP senescence-associated secretory phenotype, AD Alzheimer’s disease, COVID-19 coronavirus disease 2019, NFTs neurofibrillary tangles, APP amyloid precursor protein, PTH parathyroid hormone, FGF21 fibroblast growth factor 21, Gpld1 glycosylphosphatidylinositol-specific phospholipase D1, OCN osteocalcin, LCN2 lipocalin-2, CCL11 C-C motif chemokine ligand 11, B2M β2-microglobulin, GDF11 growth differentiation factor 11, TIMP2 tissue inhibitor of metalloproteinase 2, CSF2 granulocyte‒macrophage colony stimulating factor, PD Parkinson’s disease, α-syn α-synuclein, ALS Amyotrophic lateral sclerosis, HD Huntington’s disease, FTLD Frontotemporal lobar degeneration, ANS autonomic nervous system, RAS renin–angiotensin system. The figure was produced utilizing the applications Easy PaintTool SAI and Adobe Illustrator

The brain, serving as the central hub of the body, not only governs the activities of peripheral tissues and organs but also undergoes reciprocal influences from them, establishing a crucial network of interconnected organs and systems that uphold overall bodily function. Emerging evidence indicates that the ageing of peripheral organs contributes to brain ageing and the development of neurodegenerative diseases.^201^ Recent investigations propose that ageing constitutes a nonlinear dynamic procedure demarcated by heterogeneity among diverse organs and systems.^86,202^ This finding underscores the complex nature of ageing, indicating that interventions should be approached from a holistic perspective. Here, we aim to introduce the concept of body‒brain axes in relation to ageing and neurodegenerative diseases (Fig. 6).

#### Heart‒brain axis

At rest, the adult brain typically receives approximately 15 to 20% of the cardiac output to ensure a sufficient supply of energy and oxygen. However, the ageing process results in decreases in the ejection fraction and the portion of cardiac output allocated to the brain,^203^ as well as the contraction of the cerebral vasculature, which jointly result in chronic brain hypoperfusion (CBH).^204,205^ Additionally, the autonomic nervous system (ANS) of the heart in the elderly experiences pathological oscillations, leading to myocardial electrophysiological changes and defective activation and recovery of the myocardium, resulting in a loss of the ability to regulate the heart rate and rhythm of the heart.^206^ A recent study established the biological age (BA) of multiple human organ systems using data from the UK Biobank and revealed that cardiovascular age has the strongest influence on brain age, with a 1-year increase in the cardiovascular age increasing the brain BA by 27 days.^207^

The progression of age-related heart changes plays a pivotal role in the onset and progression of neurodegenerative diseases. AD patients often exhibit lower ejection fractions, lower cerebral blood flow velocities, and greater vascular resistance.^208,209^ CBH during ageing has been widely reported to contribute to AD pathogenesis.^210^ CBH directly enhances the synthesis and amyloidogenic processing of APP by increasing the activities of β-secretase and γ-secretase to produce Aβ.^211^ Additionally, CBH disrupts the integrity of BBB, impairing the clearance of Aβ from the brain via transcytosis. Furthermore, cerebral ischaemia and hypoxia due to CBH disrupt neuronal energy metabolism and lead to acidosis and oxidative stress, ultimately causing neuronal degeneration and cognitive impairment in an Aβ-independent manner.^212^ On the other hand, the elderly heart is prone to chronotropic insufficiency, and the inability to regulate heart rate is affected by the ageing ANS.^213^ This change is considered an early sign of PD^214^ and HD,^215^ as well as one mechanism of cognitive decline in elderly women.^216^ The measurement of vascular risk may serve as a valuable tool for the early diagnosis of patients with PD or the identification of those individuals who are at high risk, thereby confirming the potentially intricate relationship between cardiac health and PD.^217^ In addition, alterations in heart rate and the ANS are related to atrophy of the mesial temporal cortex, insula, and amygdala, as well as energy homoeostasis, which is prevalent in FTLD.^218^

#### Liver‒brain axis

The liver plays important roles in regulating metabolism and degrading metabolic wastes or poisons from the blood, thus maintaining brain and whole-body homoeostasis. Studies in humans have revealed that liver function decreases with age, as indicated by increased serum γ-glutamyl transpeptidase and alanine aminotransferase levels.^219^ Liver biopsies from older adults revealed that the degree of liver ageing is related to the severity of nonalcoholic fatty liver disease (NAFLD),^220^ which increases the brain age by approximately 4.2 years.^221^ In addition, the liver secretes neuroprotective molecules such as fibroblast growth factor 21 (FGF21) and glycosylphosphatidylinositol-specific phospholipase D1 (Gpld1), which are reported to prevent neuronal apoptosis,^222^ improve neurogenesis^223^ and even prolong the lifespan of mice.^224^ The aged liver secretes fewer neuroprotective molecules and eliminates fewer neurotoxic substances (e.g., superoxide radicals), exacerbating the accumulation of excessive oxidation products in the brain.^225^

The aged liver mainly participates in the clearance of excessive brain-derived misfolded proteins, thereby driving the pathological events of neurodegenerative diseases. The liver clears approximately 60% of circulating Aβ.^226^ However, this capacity decreases with age, which is partially attributed to the low expression of low-density lipoprotein receptor-related protein 1 (LRP-1) in hepatocytes.^227–229^ In addition, hepatic soluble epoxide hydrolase activity increases with age, decreasing the brain level of 14,15-epoxyeicosatrienoic acid, which directly binds to Aβ to prevent its deposition and indirectly enhances microglial TREM2-dependent Aβ phagocytosis, further delaying cognitive decline.^230^ In PD, brain-derived α-syn accumulates in the livers of both mice and humans; thus, the liver may participate in the clearance and detoxification of α-syn,^231^ suggesting that a decrease in aged liver function increases α-syn deposition in the brain. Furthermore, deficits in toxin clearance in aged livers increase the concentrations of circulating toxic substances, especially citrulline and ammonia, which may accelerate the onset of HD.^232^

#### Gut microbiome‒brain axis

The gut microbiome primarily regulates brain homoeostasis through the vagus nerve, endocrine system, immune system and transmission of metabolites.^233^ The microbiota and its metabolites are altered during ageing; for example, the abundance of the anti-inflammatory bacterium Faecalibacterium decreases, whereas that of proinflammatory Fusobacterium increases, leading to intestinal inflammation.^234^ The gut microbiota derived from old rats facilitates brain ageing in young rats; this effect manifests as modifications in synaptic structure and increased levels of advanced glycosylation end products (AGEs), which are markers of ageing.^235^

Microbial dysbiosis during ageing is undeniably linked to the metabolism of multiple pathogenic proteins. First, it is implicated in the release of lipopolysaccharide (LPS) and bacterial amyloid protein.^236^ The bacterial amyloid protein may induce Aβ accumulation via a prion-like seeding mechanism.^237^ In addition, LPS impedes Aβ clearance by increasing the vascular sequestration of Aβ, reducing the bulk flow of cerebrospinal fluid and impairing Aβ transport across the BBB.^238^ Moreover, LPS induces the formation of a distinct type of α-syn fibrils, similar to the pattern of wild-type α-syn fibril induction commonly observed in individuals with PD.^239^ As early as 2003, human autopsy evidence first revealed that intestinal α-syn could retrogradely diffuse from the vagal nerve to the substantia nigra and destroy dopaminergic neurons.^240^ Correspondingly, truncal vagotomy prevents the spread of α-syn from the gut to the brain, which is associated with neurodegeneration and behavioural deficits.^241,242^ Additionally, peripheral LPS promotes TDP-43 mislocalization and aggregation, contributing to TDP-43 proteinopathies in neurodegenerative disorders, such as FTLD and ALS.^243^ Second, intestinal inflammation may activate the CCAAT-enhancer-binding protein (C/EBPβ)/asparagine endopeptidase (AEP) pathway. This pathway is responsible for mediating the cleavage of APP and tau proteins, resulting in the formation of pathological fragments (e.g., APP C586 and tau N368) that promote Aβ and NFT formation, which are transmitted to the brain through the vagus nerve.^244^ In addition, activated C/EBPβ inhibits the expression of BDNF and netrin-1, leading to α-syn aggregation and dopaminergic neuronal loss.^245^ Eventually, microbial dysbiosis triggers chronic systemic inflammation, disrupting the BBB and exacerbating neuroinflammation and the progression of neurodegenerative diseases.^246–248^

#### Kidney–brain axis

The kidneys are responsible for eliminating harmful circulating substances, preventing their excessive accumulation in the brain.^249^ Kidney biopsy data from elderly individuals indicate that ageing is associated with a decline in the glomerular filtration rate.^250^ Moreover, the kidney is capable of secreting antiageing factors, such as klotho, which has been shown to enhance cognition and neural resilience. Furthermore, it has been observed that the level of kolotho decreases during the ageing process of the kidney.^251,252^ Additionally, the kidneys also release various proteins that promote brain ageing, such as kidney-associated antigen 1.^253^

To date, research on the pathogenic mechanisms of aged kidneys in neurodegenerative diseases has predominantly focused on AD and PD. The kidney serves as an organ that mediates the clearance of peripheral Aβ. Patients with CKD and animals undergoing unilateral nephrectomy exhibit elevated levels of circulating and cerebral Aβ, along with impaired cognition.^254–256^ In addition, renal insufficiency also leads to increased levels of circulating uraemic neurotoxins such as parathyroid hormone and neuropeptide Y, which adversely affect hippocampal neuronal apoptosis and the permeability of the BBB, respectively.^257^ Due to the activation of the renin-angiotensin system in aged kidneys, there is an increase in angiotensin II levels which acts on angiotensin II type 1 receptors in the substantia nigra and striatum. This induces oxidative stress and inflammation, thereby increasing the risk of PD.^258,259^

#### Lung–brain axis

The adequate delivery of oxygen to the brain heavily relies on pulmonary ventilation and gas exchange. However, lung function tends to deteriorate with age, which can be indicated by a reduction in the forced expiratory volume in one second to forced vital capacity ratio (FEV1/FVC).^260^ Furthermore, the phagocytic capacity of alveolar macrophages and neutrophils, which are responsible for pathogen clearance, diminishes in elderly individuals, thereby heightening their susceptibility to pulmonary infections.^261^ According to population surveys, poorer pulmonary function (PF) is associated with a decreased brain volume and increased white matter hyperintensity (WMH),^262^ and a 1-year increase in the lung BA increases the brain BA by 25 days.

Autopsy investigations revealed that decreased PF is associated with a greater burden of AD pathologies, including amyloid deposition and neurofibrillary tangles.^263^ The potential mechanisms may involve the induction of chronic hypoxia and subsequent activation of hypoxia-inducible factor 1 (HIF1), which in turn accelerates the production of Aβ via the overexpression of β-secretase and γ-secretase while impairing Aβ clearance through microglial dysfunction.^264^ Moreover, chronic hypoxia is thought to trigger α-syn phosphorylation and aggregation, which interacts with hypoxia-induced mitochondrial dysfunction to worsen PD progression.^265^

Additionally, a large-scale epidemiological study demonstrated that infectious diseases, including pulmonary infections, increase the risk of AD and PD dementia (PDD).^266,267^ Additionally, a special type of pathogen, *M. tuberculosis*, which primarily targets the lung, increases the risk of PD by 1.38 times. Single nucleotide polymorphisms (SNPs) in several genes, namely, LRRK2, PARK2, and PINK1, confer susceptibility to both mycobacterial infection and PD.^268^ The most common virus associated with parkinsonism is influenza. Although these viruses do not directly affect the CNS, pandemic outbreaks of influenza are associated with encephalitis with Parkinsonian features. This finding is ascribed to each of these factors inducing a significant systemic infection characterized by the production of significantly high levels of cytokines and chemokines, namely, a cytokine storm, further initiating an inflammatory cascade in the brain.^269^

The coronavirus disease 2019 (COVID-19) pandemic has emerged as the most extensive and persistent global health crisis in recorded history. The neuroinvasive nature of severe acute respiratory syndrome coronavirus 2 (SARS-CoV-2) allows it to invade the brain through both the olfactory route and the vagus nerve, which may be an important mechanism for causing clinical symptoms such as early olfactory loss, gastrointestinal and respiratory dysfunctions in COVID-19 patients.^270,271^ Autopsy evidences from COVID-19 patients directly demonstrated that SARS-CoV-2 enters the CNS partly through the olfactory mucosal-nervous milieu. This is supported by high viral RNA levels in the olfactory mucosa, and the presence of SARS-CoV spike protein in olfactory neurons.^271^ Moreover, even when respiratory testing for SARS-CoV-2 yields negative results, viral RNA can still be detected in faeces, indicating persistence and replication of SARS-CoV-2 within the gastrointestinal tract.^270^ It has been hypothesized that retrograde invasion of CNS via the vagus nerve may occur with SARS-CoV-2.^272^ Furthermore, autopsy evidence from two cases reveals immunohistochemical detection of SARS-CoV-2 in the vagus nerve fibres located on the surface of the brainstem, suggesting potential transportation of virus from lungs to brain through this pathway.^273,274^

The elderly population demonstrates a elevated susceptibility to SARS-CoV-2 infection. Epidemiological evidence indicates that individuals aged 80 and above have approximately three times higher incidence of COVID-19 compared to those aged 45 to 79, and significantly greater than individuals under the age of 24 during the initial phase of the American epidemic.^275^ Infection with SARS-CoV-2 triggers a cytokine storm, inflammation, cellular senescence, age-related immunosenescence, as well as diminished physiological reserves in the respiratory system and other organs.^276,277^ These processes are commonly associated with ageing and also implicated in the pathogenesis of neurodegenerative diseases. Furthermore, SARS-CoV-2 directly induces AD pathogenesis such as neuronal damage and amyloid processing,^278–280^ as well as PD pathogenesis including α-syn aggregation and dopaminergic neuronal loss in various models.^281^ Previous studies have demonstrated an association between COVID-19 and long-term brain atrophy and cognitive impairment in older individuals.^282,283^ The Real-time Assessment of Community Transmission (REACT) study conducted in England involving over 140,000 participants revealed that COVID-19 leads to persistent objective cognitive deficits lasting for one year or more after infection.^284^ Similarly, COVID-19 exacerbates both motor and nonmotor symptoms in PD patients, particularly urinary issues and fatigue.^285,286^ In conclusion, SARS-CoV-2 may accelerate the ageing process while increasing the risk of neurodegenerative diseases among older adults.

#### Muscle–brain axis

Muscles secrete numerous myokines that mediate bidirectional communication between the muscles and multiple organs. For example, cathepsin B and fibronectin type III domain containing protein 5 (FNDC5)/irisin have been shown to enter the brain and enhance neurogenesis and cognition.^287^ Irisin has also been revealed to increase telomerase activity to extend the lifespan.^288^ During ageing, the secretion of these myokines decreases.^289^ Additionally, a 1-year increase in the muscle BA increases the brain BA by 13 days. Therefore, a plausible speculation is that aged muscles have the potential to drive brain ageing.

Muscle ageing is a risk factor for the occurrence and development of several age-related diseases.^290^ Studies have shown that sarcopenia in elderly individuals is associated with a greater risk of AD and faster cognitive decline.^291^ However, the exact mechanisms underlying this relationship remain unclear. In light of previous studies, two potential explanations are proposed. First, the abundance of muscle-derived Aβ increases with age, potentially contributing to Aβ deposition in the brain.^292^ Second, decreased levels of myokines may account for the deterioration of cognitive function and neurodegeneration.^289^ In addition, neuromuscular junction dismantling and denervation occur in aged muscle, which are also key factors contributing to the onset of clinical symptoms and pathogenesis of ALS.^293^ In addition, a well-recognized observation in HD patients is defects in energy metabolism in skeletal muscle. mHTT affects mitochondrial complex activation and dysfunction of the mitochondrial respiratory chain in skeletal muscle, which may be markers of HD progression.^294^

#### Bone–brain axis

Bone releases cytokines such as osteocalcin (OCN) and lipocalin-2 (LCN2), as well as bone marrow-derived cells, which affect the brain. OCN promotes brain-derived neurotrophic factor (BDNF) expression and the release of inhibitory neurotransmitters to improve cognitive function. Conversely, LCN2 induces the activation of glial cells and neuroinflammation.^295^ During ageing, bone support is diminished due to decreased OCN levels, as well as increased LCN2 and sclerostin levels. Moreover, age-related brain atrophy and ventricular enlargement have been linked to osteoporosis, further emphasizing the impact of aged bone on brain ageing.^296^

Osteoporosis may accelerate atrophy of the entorhinal cortex and hippocampus, increasing the risk of AD by 1.27 times.^297^ Accordingly, in a study of a large number of postmenopausal women, osteoporosis increased the risk of PD by 1.4 times.^298^ Even if no obvious evidence for the relationship between osteoporosis and HD is available, bone mineral density is significantly lower in pre-HD carriers than in healthy controls.^299^ Additionally, osteoblasts have been reported to produce Aβ, this might be involved in the development of AD. Transplantation of bone marrow mesenchymal stem cells upregulated beclin-1 expression, increasing autophagy in the hippocampus to clear Aβ.^300^ Furthermore, changes in bone-derived cytokines during ageing may also be implicated in several neurodegenerative diseases.^301^ For example, OCN decreases the Aβ load, increases glycolysis in microglia and astrocytes,^302^ and ameliorates motor deficits and dopaminergic neuronal loss in PD mice.^303^ LCN2 and sclerostin aggravate neuroinflammation and abolish synaptic plasticity,^304,305^ thereby accelerating the progression of AD, PD and ALS.^306–308^

#### Blood‒brain axis

The blood circulation connects the brain and each organ of the body, thus collecting pro-ageing and antiageing factors derived from various organs or systems. Systemic factors in the blood can directly cross the BBB or blood–cerebrospinal fluid barrier, or indirectly transduce signals to target neurons, astrocytes, microglia, and other targets to regulate brain function.^309^ Exposing a young mouse to plasma from old mice impairs synaptic plasticity, neurogenesis and cognition,^310,311^ suggesting that aged blood contributes to brain ageing.

A variety of complex components of the circulatory system are associated with neurodegenerative diseases. Blood-derived Aβ has been found to enter the brain, inducing homoeostasis disorders and AD-related pathology.^312^ Platelets, which are responsible for approximately 90% of circulating Aβ,^313^ are overactivated with ageing^314,315^ and are reported to release more Aβ and subsequently induce Aβ deposition in the brain and cognitive impairment.^316^ Additionally, serum albumin, which is responsible for adhering to and transporting Aβ, is inversely associated with Aβ deposition in the brain.^317^ Serum albumin levels decrease with age,^318^ possibly increasing Aβ deposition in the brain, as albumin is able to sequester Aβ from the blood.^319^

Emerging systemic factors in the blood are associated with neurodegenerative pathological events. During ageing, the levels of pro-ageing factors such as C-C motif chemokine ligand 11 (CCL11) and β2-microglobulin (B2M) in the blood gradually increase, which damages synapse, neurogenesis and cognition.^310,320,321^ In contrast, the levels of antiageing factors, such as GDF11, tissue inhibitor of metalloproteinase 2 (TIMP2) and granulocyte‒macrophage colony stimulating factor (CSF2), are reduced. These factors are linked to improved microglial phagocytosis and neurogenesis and reduce the Aβ load, thus enhancing cognition.^72,322–324^ Similarly, in PD animal models, GDF11 overexpression inhibits oxidative stress, cell senescence and apoptosis of dopaminergic neurons.^325^

#### Immune–brain axis

The immune system is responsible for protecting the host from endogenous and exogenous antigens to maintain body homoeostasis.^326^ Immunosenescence is characterized by dysfunctions in monocyte and neutrophil phagocytosis, decreased numbers of naive T cells, increased numbers of memory T cells,^327^ and increased SASP secretion by these cells,^327,328^ thereby weakening the immune response to foreign antigens. Notably, the selective induction of immune cell ageing increases the levels of the ageing markers p16 and p21 in multiple organs, including the brain,^328^ thereby highlighting the crucial roles played by the immune system in the process of ageing.

An intricate correlation has been identified between immunosenescence and neurodegenerative diseases. The phagocytic capacity of aged peripheral myeloid cells decreases during ageing and in AD,^329,330^ and adaptive immune cells produce a disordered antibody profile, leading to impaired Aβ clearance.^331^ A longer leukocyte telomere length is related to a greater hippocampal volume and lower WMHs, predicting a lower AD risk.^332^ Additionally, during abnormal immune ageing, the typical age-associated shift towards senescence in the CD8^+^ T-cell population may be attenuated, resulting in a heightened immune response to misfolded α-syn and thereby increasing the risk of PD.^333^ Similarly, in a single-centre, retrospective study, increased numbers of senescent and late memory T and B lymphocytes were characteristic of faster progressing ALS.^334^ Despite the lack of direct evidence, alterations in immune-regulatory factors, such as elevated levels of interleukin (IL)-6 and monocyte activation, observed in the plasma of early HD patients or even prior to HD onset, provide support for early activation of the immune system in the periphery. This parallels the activation seen in the CNS, suggesting potential crosstalk between the periphery and the CNS may exist.^335^ Moreover, in elderly patients with FTLD, genes associated with phagosomes and lysosomes in peripheral blood mononuclear cells are downregulated,^336^ indicating a potential link between the peripheral immune system and FTLD. Additionally, peripheral inflammatory markers, including IL-2, IL-17A, IL-12p70, tumour necrosis superfamily member 8 (TNFRSF8) and tumour necrosis factor (TNF)-α, are associated with neurodegeneration in individuals with FTLD, such as brain atrophy and abnormal metabolism, which are mainly distributed in frontal‒temporal regions.^337^ Subsequent intensive research even suggested that plasma IL-6 and TNF-α levels may be positively correlated with the rate of cognitive decline.^338^

Immunosenescence renders elderly individuals more susceptible to infection by specific pathogens and neurotropic virus, which are confirmed to increase the risk of neurodegenerative diseases, such as pathogens causing periodontitis,^339,340^ herpes simplex virus 1 and hepatitis C virus.^341^ Neurotropic virus directly invade CNS through BBB and peripheral nervous system, accompanied by the SASP, activation of microglia and astrocytes, neuroinflammation, jointly promoting neurodegeneration and cognitive impairment.^342–344^

Finally, the systemic chronic inflammation resulting from immunosenescence and non-neurotropic viral infection compels immune cells in the peripheral blood to penetrate the blood-brain barrier (BBB) and choroid plexus and enter the brain,^345,346^ Additionally, immune cells originating from the cranial bone marrow migrate to the meninges,^347^ thereby promoting neuroinflammation and neuronal dysfunction while inhibiting neurogenesis. Consequently, this cascade of events triggers neurodegenerative diseases.^201,348^

#### Endocrine–brain axis

The integration of body functions within the endocrine system is facilitated by hormones Many hormones, including gonadal hormones, glucocorticoids (GCs), thyroid hormones and insulin, are prominently involved in brain activities, including synaptic connections, energy metabolism, neurogenesis and glycometabolism. During the ageing process, these hormones are perturbed due to dysregulation of the major endocrine axes, including the hypothalamic–pituitary–gonadal (HPG) axis, hypothalamic–pituitary–adrenal (HPA) axis and hypothalamic–pituitary–thyroid (HPT) axis, leading to age-related diseases.^349^ Decreased oestrogen levels are associated with shorter telomere lengths in postmenopausal women, potentially shortening longevity.^350^ Moreover, elderly people with type 2 diabetes have an average increase of 4.6 years in the brain age gap estimation (brainAGE).^351^ Furthermore, insulin resistance has been found to reduce life expectancy through epigenetic clocks.^352^

Ageing-related changes in the endocrine system are linked to neurodegenerative diseases, partly because of their impacts on pathological proteins. The elevated level of follicle-stimulating hormone (FSH) directly promotes the accumulation of Aβ and hyperphosphorylated tau in the hippocampus via the C/EBPβ-δ secretase pathway, leading to neuronal apoptosis, synaptic damage and spatial learning deficits.^353^ Additionally, oestrogen deficiency is more likely to induce the accumulation of Aβ and α-syn in the brain.^354,355^ In addition, insulin resistance affects the clearance of Aβ, the hyperphosphorylation of tau, and glucose metabolism and enhances the aberrant expression of α-syn.^356,357^ Moreover, hyperglycaemia aggravates the phosphorylation and aggregation of α-syn, neuroinflammation and dopaminergic neuronal loss in PD mice.^358^

In addition to perturbations in protein homoeostasis, endocrine system disorders contribute to the pathogenesis of neurodegenerative diseases through other molecular mechanisms.^359^ For example, the levels of tetraiodothyronine (T4) and triiodothyronine (T3) decrease in elderly individuals, resulting in AD-related reducion in blood perfusion in memory-related regions and decrease in energy supply to the CNS due to low glucose metabolism.^360,361^ Moreover, elevated levels of circulating luteinizing hormone (LH) during ageing process impair BDNF expression and synaptic plasticity.^362^ Additionally, insulin resistance induces the loss and apoptosis of dopaminergic neurons in individuals with PD by inhibiting the neuroprotective protein kinase B (Akt) pathway,^363^ and the severity of insulin resistance correlates with that of nonmotor disorders in patients with PD.^364^ Moreover, several studies have shown that oestrogen disturbances and increased GCs elicit mitochondrial dysfunction, ultimately leading to neuronal and cognitive impairment.^352,365^ The persistent and chronic elevation in GC levels has a detrimental impact on the functionality of the glucocorticoid receptor (GR). Microglial GR has a crucial role in attenuating microglial cell activation and reducing dopaminergic degeneration. GCs are also known to regulate BBB permeability, affecting the infiltration of cytotoxic molecules and resulting in increased vulnerability of dopamine neurons in PD.^366^ Ultimately, excessive ACTH leads to activation and hypertrophy of the adrenal cortex in aged HD mice, resulting in elevated cortisol levels,^367^ that may contribute to impaired glucose metabolism,^368^ skeletal muscle atrophy, and weight loss.^369^

Except for the above body-brain axes, high fat and obesity are widely known to have negative effects on ageing and AD.^370,371^ Epidemiological study has found that midlife central obesity increases risk of dementia as high as 3.6 times,^372^ possibly by activating systemic inflammation, thus worsening astrogliosis, microgliosis, neuroinflammation and Aβ pathology.^373,374^ Therefore, healthy lifestyles, such as regular physical activity and healthy diet, are associated with weigh loss and reduced the risk of dementia by 0.68 times.^375,376^

## Antiageing strategies for neurodegenerative diseases: preclinical studies

Ageing and neurodegenerative diseases are progressive and currently irreversible. Throughout the process of ageing, the accumulation of damage resulting from aberrant responses to both internal and external changes ultimately leads to functional decline, chronic diseases, and eventually mortality.^85^ The objectives of antiageing interventions primarily focus on the delay, prevention, or even reversal of ageing effects. However, achieving these goals poses challenges due to the limitations of current technological methodologies. Consequently, current preclinical studies in antiageing can only decelerate or mitigate the ageing process, with some studies partially alleviating the effects of ageing and thereby preventing the onset or delaying the progression of neurodegenerative diseases.

From a complex systems perspective on ageing, it exerts systemic effects across multiple levels and dimensions within the body. Given the intricate and systemic nature of the ageing process, it is imperative to implement multiple interventional measures simultaneously rather than relying on a single measure in order to achieve more effective outcomes in antiageing interventions. Furthermore, considering that the ageing process and comorbidities expedite the progression of neurodegeneration throughout the entire body system, merely clearing misfolded pathogenic proteins and providing symptomatic treatment are insufficient to halt disease progression or prevent the onset of neurodegenerative disorders. In addition to focusing on neurodegenerative disease-related mechanisms within the brain, it is essential to broaden our attention to the role of systemic risk factors of neurodegenerative diseases throughout the ageing process. Consequently, the need for individualized management of risk factors and comorbidities during the progression of neurodegenerative diseases is evident, alongside the implementation of person-centred care models and social support.^377^

**Fig. 7:**
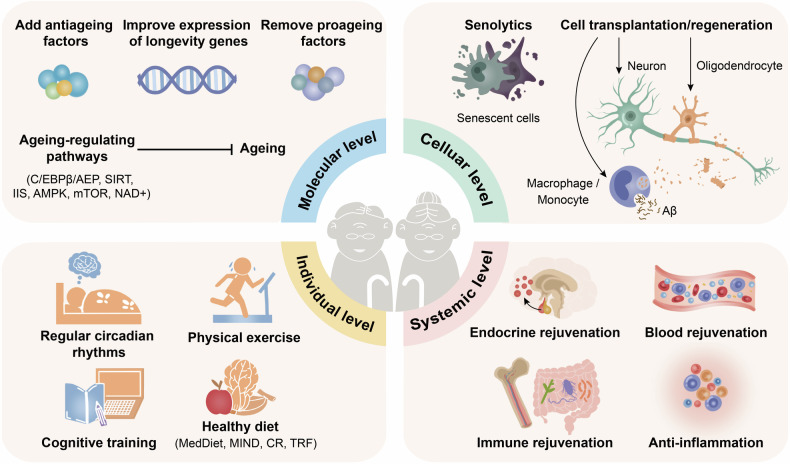
Holistic antiageing strategies. Antiageing strategies need to be implemented systemically at the molecular, cellular, systemic and individual levels. This holistic approach shows promise for preventing brain ageing and treating neurodegenerative diseases. C/EBPβ/AEP CCAAT-enhancer-binding protein/asparagine endopeptidase, IIS insulin/IGF-1 signalling, AMPK 5’-monophosphate-activated protein kinase, SIRT sirtuin, mTOR mammalian target of rapamycin, NAD^+^ nicotinamide adenine dinucleotide, Aβ amyloid-β, CR caloric restriction, MedDiet Mediterranean diet, MIND Mediterranean–DASH intervention for neurodegenerative delay, TRF time-restricted feeding. The figure was produced utilizing the applications Easy PaintTool SAI and Adobe Illustrator

Therefore, the antiageing interventions discussed herein primarily address the systemic effects of ageing. Targeting the entire system rather than focusing on a single element is considered the optimal approach to interfere with ageing and mitigate neurodegeneration.^378^ We will elaborate on potential systemic antiageing interventions at the molecular, cell, organ/system, and organism levels, to provide an efficient and insightful approach for treating neurodegenerative diseases (Fig. 7).

### Antiageing strategies at the molecular level

#### Supplementation with antiageing molecules

The selective supplementation of blood-derived antiageing factors holds promise for revitalizing the aged brain.^379^ Studies using aged animal models have verified that elevating the levels of circulating OCN, GDF11, and FNDC5 is associated with an augmentation in BDNF levels and enhancement of neurogenesis.^380–382^ Additionally, irisin reduces the formation of pathologic α-syn, prevents the loss of dopaminergic neurons, and improves α-syn-induced motor deficits.^383^ According to recent reports, replenishing clusterin and platelet factor 4 (PF4) ameliorates neuroinflammation,^384,385^ and oleoylethanolamide (OEA) enhances microglial phagocytosis,^386^ all of shese contribute to cognitive improvement and neuroprotection. Moreover, TIMP2 in umbilical cord plasma enhances synaptic plasticity and improves hippocampus-dependent cognition in aged mice.^322^ In in vitro experiments, thrombospondin-4 (THBS4) and SPARC-like protein 1 (SPARCL1) act directly on neurons to stimulate synapse formation and enhance synaptic responses.^387^ Despite the impermeability of the BBB, peripherally administered α-klotho protein fragments induce neural resilience and N-methyl-D-aspartic acid receptor (NMDAR)-dependent synaptic plasticity in PD animal models.^251^ Compared with monotherapy, combination therapy with transforming growth factor-β receptor I (ALK5) inhibitors and oxytocin synergistically reverses the ageing phenotype in multiple organs,^388^ indicating that simultaneous intervention of multiple antiageing pathways yields better outcomes.

Increasing the expression of longevity genes is also promising in inhibiting ageing in animal models. Directly increasing klotho expression in the brain alleviates the loss of neurons and synapses related to memory in senescence-accelerated mice.^389^ Similarly, the cognitive benefits of klotho have recently been validated in aged nonhuman primates.^252^ Furthermore, an extension of the lifespan is observed when the telomerase reverse transcriptase (TERT) gene is overexpressed.^390^ Overall, the use of antiageing factors increases the treatment efficacy and minimizes adverse responses. Thus, identification of supplementary effective antiageing components in combination therapies is an essential objective for further research.

#### Elimination of pro-ageing molecules

The removal of circulating pro-ageing factors also holds the potential to revitalize the brain in animal studies.^391^ Neutralizing antibody treatment or gene editing to lower circulating levels of CCL11 and B2M alleviates neuroinflammation and age-related cognitive decline.^320,392^ Additionally, vascular cell adhesion molecule 1 (VCAM1) and acid sphingomyelinase (ASM) play a curcial role in destroying the cerebrovascular system,^393,394^ while cyclophilin A (CyPA) decreases the levels of synapse-related proteins such as the NMDAR subunit NR2B and synaptophysin.^395^ Reducing the levels of these factors in aged plasma partially mitigates their detrimental effects on young brains. The systemic factors in the blood are classified and summarized in Table 1.

**Table 1:** Systemic ageing-related molecules

	Molecule	Animal model	Cell source	Function	References
Pro-ageing	CCL11	Aged mice	Epithelial cells and macrophages	Inhibits neurogenesis and impairs cognition	^310^
	β2M	Aged mice	Nearly all nucleated cells	Promotes hippocampus-dependent cognitive dysfunction and impairs neurogenesis	^320^
	VCAM1	Aged mice	Endothelial cells	Increases microglial reactivity and cognitive deficits	^393^
	CyPA	Aged mice	Mainly brain, lung, kidney and macrophages	Decreases synapse-related protein expression and cognitive function	^395^
	ASM	Aged mice	Brain, immune cells, heart, spleen, muscle and liver	Causes endothelial cell death, reduces BBB integrity, and results in neuronal dysfunction	^394^
	SASP	Aged mice	Senescent cells	Causes inflammation throughout the body and drives ageing in other tissues	^429^
	LCN2	MPTP-induced PD mice, pentobarbital-induced AD mice	Mainly neutrophils, macrophages, and adipose tissue	Accelerates astrocyte senescence and AD and PD progression	^306,308^
Antiageing	GDF11	Aged mice, MPTP-induced PD mice	Mainly the brain, adrenal gland, soft tissue and testis	Enhances neurogenesis and improves the vasculature and neuronal activity/plasticity	^325,381^
	THBS4 and SPARCL1	Serum	Extracellular matrix	Enhances synaptic responses and increases synapse numbers	^387^
	CLU	mThy-1-hAPP751_V171I, KM670/671NL_ mice	Mainly hepatocytes and cardiomyocytes	Downregulates interferon and cytokine signalling pathways to reduce neuroinflammation	^384^
	FGF21	Aged mice	Mainly the liver, pancreas and adipose tissue	Suppresses the oxidative stress response, reduces brain cell damage and improves cognition	^222^
	Oxytocin+ALK5i	Aged mice	Pituitary gland secretes OT	Enhances neurogenesis, reduces neuroinflammation, and improves cognition	^388^
	Gdld1	Aged mice	Liver	Ameliorates impairments in neurogenesis and cognition	^223^
	FNDC5/irisin	APP/PS1 mice, mice with preformed α-syn fibrils	Mainly muscle	Reverses synaptic failure and memory impairment, reduces pathologic α-syn levels	^287,383^
	Osteocalcin	Aged mice, APP/PS1 mice, 6-OHDA-induced PD mice	Bone	Improves memory and decreases anxiety-like behaviours, the Aβ load and dopaminergic neuronal loss	^302,303,380^
	Platelet factor 4	Aged mice	Platelet	Reduces neuroinflammation and improves synaptic-related markers, immune responses and cognition	^385^
	Oleoylethanolamide	5xFAD mice	Mainly small intestine epithelial cells	Enhances microglial Aβ clearance and reverses the dysregulation of lipid profiles and cognitive impairments	^386^
	Oestrogen	Sprague‒Dawley rats	Mainly the ovary	Upregulates telomerase activity and TERT mRNA expression	^464^
	Growth hormone-releasing hormone	Humans	Hypothalamus	Improves cognition in older adults and ameliorates MCI	^532^
	Gonadotropin-releasing hormone	Aged mice	Hypothalamus	Amends ageing-impaired neurogenesis and decelerates ageing	^463^
	TERT	Aged mice	Mainly germ cells and stem cells	Prolongs the lifespan and reverses ageing-related phenotypes	^390^
	Klotho	SAMP8 mice, nonhuman primates and transgenic α-syn mice	Mainly the kidney and brain	Improves ageing-related impairments in cognition and neural resilience, as well as decreases oxidative stress	^251,252,389^
	TIMP2	NSG mice	Mainly the reproductive system and bladder	Increases synaptic plasticity and hippocampus-dependent cognition in aged mice	^322^
	CSF2	APP/PS1 mice	Mainly macrophages, T cells, mast cells, natural killer cells, endothelial cells, and fibroblasts	Reduces brain amyloidosis, maintains synaptic integrity and improves cognition	^324^

*CCL11* C-C motif chemokine ligand 11, *β2M* β2-microglobulin, *VCAM1* vascular cell adhesion molecule-1, *CyPA* cyclophilin A, *ASM* acid sphingomyelinase, *BBB* blood-brain barrier, *SASP* senescence-associated secretory phenotype, *GDF11* growth differentiation factor 11, *THBS4* thrombospondin-4, *SPARCL1* SPARC-like protein 1, *APP* amyloid precursor protein, *CLU* clusterin, *PS1* presenilin 1, *FGF21* fibroblast growth factor 21, *ALK5i* TGFβ receptor I receptor kinase inhibitor, *OT* oxytocin, *Gpld1* glycosylphosphatidylinositol-specific phospholipase D1, *FNDC5* fibronectin type III domain containing protein 5, *AD* Alzheimer’s disease, *TERT* telomerase reverse transcriptase, *Aβ* amyloid-β, *MCI* mild cognitive impairment, *SAMP8 mice* senescence-accelerated mouse-prone 8 mice, *TIMP2* tissue inhibitor of metalloproteinase 2, *NSG mice* NOD-Prkdcscid-Il2rgtm1 mice, *FAD* familial AD, *CSF2* granulocyte-macrophage colony-stimulating factor, *PD* Parkinson’s disease, *6-OHDA* 6-hydroxydopamine, *MPTP* 1-methyl-4-phenyl-1,2,3,6-tetrahydropyridine, *α-syn* α-synuclein, *LCN2* lipocalin-2

#### Targeting ageing-related signalling pathways

To date, numerous ageing-regulating pathways, including the IIS, glucagon-like peptide-1 (GLP-1), AMPK, sirtuin, mTOR and NAD^+^ pathways, have been identified.^126,396,397^ The expression of the transcription factor C/EBPβ has been found to increase with age, resulting in the activation of AEP (also known as δ-secretase) transcription and ultimately leading to excitatory neurotoxicity and a shortened lifespan.^398^ This pathway has also been implicated in AD and PD.^399,400^

Drugs that target the above pathways have been confirmed to have antiageing effects.^401^ AMPK activators and the mTOR signalling pathway inhibitor rapamycin have considerable ramifications for longevity and managing age-related illnesses.^58,402–404^ Metformin, an effective drug, has been shown to improve hallmarks of ageing, such as DNA repair and imbalanced protein homoeostasis.^405^ Following a metformin intervention spanning up to 3.3 years in middle-aged and elderly cynophagus monkeys, the overall degree of ageing was comprehensively assessed by high-throughput omics techniques. Surprisingly, metformin systematically improves the characteristics and hallmarks of ageing, lowering epigenetic age by up to 6.1 years (frontal lobe). More notably, metformin had a prominent effect on the ageing brain, which was reflected in increasing cognitive resilience and brain reserve, rejuvenation of the transcriptomics of nerve cells, as well as in the autonomous alleviation of neuronal senescence.^61^ GLP-1 is an incretin hormone that targets insulin signalling to lower sugar levels, and glucagon-like peptide-1 receptor agonists (GLP-1 RAs) are widely used to alleviate oxidative stress, chronic inflammation, cellular senescence and apoptosis.^406^ GLP-1 RAs reverse transcriptomic ageing signatures in multiple major brain cell types, including glial cells and neurovascular cells.^407^ Intriguingly, a cocktail treatment containing rapamycin, acarbose and phenylbutyrate interferes with multiple antiageing pathways, achieving greater efficacy in delaying ageing in mice.^408^ Through RNA sequencing, the drug cocktail subsequently downregulates the transcriptomic profiles of three major ageing pathways, namely, the mTOR, IIS and histone deacetylase binding pathways. With effects on these factors, the drug cocktail effectively inhibits biological processes that contribute to ageing, such as DNA damage, inflammation, and cell senescence, while simultaneously promoting a significant increase in autophagy.^409^

Antiageing drugs have favourable effects on neurodegenerative diseases. Age-dependent NAD^+^ depletion and impaired mitophagy downstream may exacerbate the progression of these diseases. NAD^+^ augmentation ameliorates both Aβ and p-tau pathologies and neuroinflammation.^125^ Supplementation with the NAD^+^ precursor NAM rescues mitochondrial defects and behavioural impairments in AD models^410^ but suppresses dopaminergic neurodegeneration in a Drosophila PD model.^411^ Additionally, another NAD^+^ precursor, NR, improves HD motor and molecular phenotypes, possibly through the activation of SIRT1-PGC-1α and SIRT3-related pathways.^412^ These pathways are also involved in ALS pathogenesis.^413^ Likewise, SIRT1 inhibits Aβ production and neuroinflammation and prevents neuronal apoptosis in AD models.^414^ Correspondingly, the sirtuin family adjusts pathways related to mitochondrial biogenesis and dysfunction, oxidative stress and α-syn aggregation in individuals with PD.^415^ Unexpectedly, SIRT regulates TDP-43 posttranslational modifications, reducing the aggregation propensity via deacetylation,^123^ and further in vivo experiments are needed to confirm its effectiveness. Rapamycin may be beneficial in the early stages of AD, but it aggravates AD pathology as the lysosomal degradative capacity of the brain deteriorates.^416,417^ In PD mice, rapamycin activates autophagy to inhibit ferroptosis, exerting a beneficial effect on behavioural symptoms and the loss of dopaminergic neurons in the substantia nigra pars compacta.^418^ Due to enhanced autophagy, rapamycin can ameliorate the changes in locomotor performance and reduce HTT aggregation in the brains of Drosophila HD models,^419^ while rapamycin has also been shown to be neuroprotective in ALS and FTD.^420^ Previous studies have revealed that AD/PD and type 2 diabetes mellitus share some pathological commonalities, strongly suggesting the role of antidiabetic drugs in treating neurodegenerative diseases,^421,422^ and the most common high-profile drugs are metformin and GLP-1 RAs. Metformin reduces the burden and toxicity of pathological proteins, including Aβ, p-tau, α-syn and HTT, protects neurons, and enhances cognitive and motor function in multiple animal models.^423,424^ Notably, metformin also improves behaviour and pathology in ALS/FTD mice.^425^ In contrast, metformin has adverse effects in some studies. According to multiple preclinical studies, GLP-1 RAs reduce amyloid deposition and glial cell activation and stimulate synaptic neurotransmitter release to induce long-term potentiation (LTP) in AD models.^426^ However, GLP-1 RAs enhance motor performance and dopamine signalling and inhibit the aggregation of α-syn in PD models, possibly by regulating the Akt pathway.^423,427^ An inspiring result has shown that a drug cocktail restores cognitive impairment, neuroinflammation, and Aβ aggregation while enhancing autophagy and synaptic integrity in AD mice, especially in females.^428^ This result highlights the efficacy of multi-target antiageing interventions.

### Antiageing strategies at the cellular level

Senolytics kill senescent cells precisely and target the SASP to delay or alleviate tissue disorders, with promising prospects for antiageing applications.^429^ The eradication of senescent cells in pre-ageing mice partially counteracts the age-related functional decline and extends the lifespan by up to 35%.^63^ The delivery of senolytics in AD mice reduces the Aβ load and the levels of proinflammatory factors, thereby enhancing cognitive funciton.^430^ Additionally, senolytic and senomorphic secondary metabolites inhibit α-syn aggregation and prolong the healthspan in PD models.^431^ Despite all the benefits, the utility of senolytics is not undisputed. First, they lack specificity for the targeted elimination of senescent cells. Second, using senolytics too early results in stem cell depletion, accelerating the ageing process, whereas their delayed use may affect their effectiveness. Additionally, controversies exist over which type and how many senescent cells should be removed for optimal efficacy. Ageing is a global process affecting all tissues, organs, and cells within the body. In the context of neurodegenerative diseases, the elimination of senescent cells is not always beneficial. For cell types possessing regenerative capabilities, selectively removing senescent cells (such as microglia) can diminish neuroinflammation levels, thereby serving a neuroprotective function. However, for terminally differentiated cells like neurons, removal of senescent ones may exacerbate ageing phenotypes and neurodegenerative conditions due to the absence of available replacement cells to maintain functionality.

Cell transplantation or regeneration has the potential to reverse ageing and decrease susceptibility to neurodegenerative diseases. The administration of muscle-derived stem/progenitor cells from young mice to progeroid mice results in muscle regeneration and a significant lifespan extension.^432^ In addition, macrophages remove myelin debris to promote myelin regeneration and reverse the age-related dedifferentiation of oligodendrocytes,^433^ whereas monocyte enrichment increases monocyte infiltration in the brain, resulting in the engulfment of Aβ deposits.^434^ Furthermore, cotransplantation of midbrain dopaminergic neurons and autologous regulatory T cells into PD rats improves the survival of dopaminergic neurons and motor function.^435^ Encouragingly, the conversion of astrocytes into neurons via NeuronD1 may compensate for neurodegeneration during ageing and AD.^436^

Despite the potential antiageing effects, stem cell transplantation is hindered by numerous adverse effects that impede its clinical application. These include rejection of allogeneic cells by host immune cells,^437^ graft-versus-host disease,^438^ infections resulting from long-term suppression of the immune system, and tumorigenicity.^439^ Alternatively, extracellular vesicles derived from stem cells offer a more feasible approach for application. Studies have demonstrated that extracellular vesicles derived from adipose mesenchymal stem cells and umbilical cord mesenchymal stem cells can improve a wide range of age-related phenotypes and delay the ageing process in aged mice.^440–442^ Neural stem cell-derived extracellular vesicles (NSC-EVs) are abundant in specific miRNAs which exert favourable effects on slowing down ageing and neurodegenerative diseases.^443^ NSC-EVs have been shown to inhibit neuroinflammation and ameliorate pathological events and behaviours in AD mice and PD models.^444,445^ A recent investigation revealed that the administration of extracellular vesicles purified from the plasma of young mice enhanced mitochondrial function, partially restored the proteome, metabolism, and physiological capabilities of multiple organs, resulting in a noteworthy 12.4% increase in the median lifespan of mice.^446^

### Antiageing strategies at the systemic level

The circulatory, immune and endocrine systems, which are tightly interconnected with the entire body, are promising targets for systemic antiageing interventions, which are expected to achieve comprehensive rejuvenation.

#### Rejuvenation of the blood

Numerous animal experiments have shown that young blood prolongs the BA of aged recipients via epigenetic remodelling^447^ and rejuvenates various organs, including the brain.^379,448^ The possible mechanisms include the activation of cAMP response element binding protein (CREB) in the hippocampus and canonical neuroprotective mechanisms.^449,450^ After organ transplantation from old to young individuals, the transplanted organs remain functional after the maximum lifespan of the original donor, indicating the rejuvenation of the aged organs in a young systemic environment.^451^ Injections of human umbilical cord plasma into elderly individuals reduce the epigenetic age by 0.82 years and improve several clinical parameters, such as creatinine levels and the glomerular filtration rate.^452^ Blood rejuvenation also has the potential to treat AD. Whole blood replacement mainly lowers soluble Aβ levels in the blood and Aβ deposits in the brains of aged mice and markedly improves spatial memory.^453^ Blood rejuvenation allows the delivery of a more comprehensive range of antiageing factors, targeting multiple markers of ageing in combination. This approach may lead to greater efficacy, although it requires a sufficient blood supply and potentially causes adverse reactions.

#### Rejuvenation of the systemic immune system

Immunosenescence is a key driver of systemic ageing and could be a valuable target for antiageing interventions. Research suggests that transplantation of young bone marrow plays a positive role in preserving synaptic connections and cognitive manifestations in aged mice,^454^ increasing the maximum lifespan by 30%.^455^ Bone marrow-derived microglia are involved in clearing Aβ and have potential for AD therapy.^456^ A recent study revealed that the transplantation of bone marrow stem cells from young AD mice to old AD mice reversed the expression of ageing-related differentially expressed genes (DEGs), compromised phagocytosis of monocytes, and cognitive impairment.^457^ Correspondingly, the transplantation of bone marrow from WT mice into HD mice partially alleviates motor deficits, elevates cortical synaptic levels and reduces serum inflammatory factors, including IL-6, IL-10, CXC chemokine ligand 1, and IFN-γ.^458^

However, the clinical use of bone marrow transplantation is limited because of the shortage of young bone marrow donors and rejection after transplantation. As such, a more feasible approach is to rejuvenate the aged gut microbiota. Transplantation of the gut microbiota from young mice to aged mice reverses immunosenescence and neuroinflammation and improves hippocampal neurogenesis, behaviour and cognition.^75,459^ Additionally, metabolomics and gene regulation patterns in the brains of old mice switch to a young phenotype.^75^ Strikingly, transplantation of the gut microbiota from wild-type mice to AD mice alleviates the Aβ load, neurofibrillary tangles and glial reactivity.^460^ Similarly, transplantation of healthy human faecal microbiota protects integrity of the BBB and reduces the entry of gut-derived harmful substances into the brain, thereby alleviating neuroinflammation and neurodegeneration in PD mice.^461^ Small-scale research has verified the safety and efficacy of faecal microbiota transplantation, as reflected in improvements in motor and nonmotor symptoms in PD patients.^462^ These studies highlight the importance of rejuvenating the aged immune system for a healthy lifespan and for the treatment of neurodegenerative diseases.

#### Rejuvenation of the endocrine system

Gonadotropin-releasing hormone ameliorates neurogenesis and decelerates the ageing process in mice.^463^ Additionally, oestrogen supplementation in ovariectomized rats is capable of rejuvenating multiple organs by increasing telomerase activity and TERT expression,^464^ but the utility of oestrogen replacement therapy in the general population is still debatable.^465^

Hormone therapy has achieved some progress in the treatment of neurodegenerative diseases. A GHRH analogue and exogenous insulin-like growth factor-2 (IGF2) are neuroprotective.^466^ IGF2 stimulates neurogenesis and synaptogenesis and enhances cognition in AD models.^467^ Moreover, oestrogen signalling is involved in AD pathogenesis. Oestrogen receptor α (ERα) and oestrogen receptor β (ERβ) are widely distributed in the CNS, and their overexpression protects neurons from glutamatergic excitotoxicity and Aβ toxicity.^468^ Moreover, oestrogen regulates transcription factors related to inflammation and oxidative stress, such as nuclear factor kappa-B (NF-κB) and nuclear factor erythroid 2-related factor 2 (Nrf2), to alleviate neuroinflammation and other AD pathologies.^469^ In PD models, oestrogen enhances the neuroprotective function of astrocytes, reduces the vulnerability of substantia nigra dopaminergic neurons,^470^ and improves motor deficits.^471^

#### Anti-inflammation

Ageing is accompanied by long-term chronic low-grade inflammation, namely, inflammageing. Inflammation is implicated in various pathways and processes associated with ageing, including immunosenescence, oxidative stress, metabolic dysregulation, cellular senescence, and other critical events of the ageing process.^37^ Therefore, modulating the body’s inflammatory balance is expected to increase longevity and reverse or mitigate age-related disease processes.^472–474^ In mouse models of accelerated ageing, inflammation is exacerbated by the overactivation of NF-κB, thereby blocking this pathway and consequently conferring longevity.^475^ A recent investigation demonstrates that neutralization of the inflammatory cytokine IL-11 ameliorates age-related metabolic disorders, enhances overall physiological function, and extends the average lifespan of mice by 24.9%.^476^ Nonsteroidal anti-inflammatory drugs (NSAIDs), such as ibuprofen, reduce neuroinflammation and the senescent cell burden, resulting in significant improvements in cognitive function in premature mice.^477^ Furthermore, aspirin has been confirmed to extend the lifespan of *Drosophila melanogaster* and mice in a sex-dependent manner.^478,479^ Additionally, plant extracts, such as resveratrol and ginkgo biloba extract, have been demonstrated to effectively delay the ageing process of animal organs, i.e. the liver and ovarian.^480,481^

Targeting age-related inflammation is beneficial for the management of neurodegenerative diseases.^482^ NSAIDs are involved in the prevention and treatment of AD by activating peroxisome proliferator-activated receptor gamma (PPARgamma) to reduce the neurotoxicity of microglia and monocytes and astrocyte activation.^483^ However, NSAIDs have an antagonistic effect on PD that is beneficial through the modulation of neuroinflammation but detrimental through the inhibition of neuroprotective prostacyclin (PGI2) and accentuation of proinflammatory leukotrienes (LTs).^484^ Furthermore, a meta-analysis indicated that NSAIDs are associated with a decreased risk for the development of ALS.^485^

### Antiageing strategies at the individual level

Estimates from basic studies indicate that individuals may delay brain ageing and reduce age through cognitive training, the regulation of circadian rhythms, diet and exercise. An overlap between the mechanisms of these lifestyle factors has been observed. Cognitive training enhances functional connectivity to attenuate the decline in overall memory.^486^ Time-restricted feeding (TRF), a healthy diet, extends the Drosophila lifespan and delays the onset of ageing markers in the muscles and gut by stimulating circadian-regulated autophagy.^487^ Normalization of the dysregulated circadian clock may decelerate brain ageing.^488^ Likewise, CR triples the median and maximal remaining lifespans of progeroid mice, strongly retarding numerous aspects of accelerated ageing. In mice subjected to CR, 50% more neurons and full motor function are retained.^489^ Additionally, single-cell transcriptome sequencing revealed that long-term exercise significantly reduces the degree of pantissue ageing, remodels the structures and functions of multiple organs and tissues, and enhances cognitive function in aged mice.^490^

In parallel, these beneficial lifestyles are also adapted to confer a cognitive reserve, slow progressive neurodegeneration, and ameliorate pathological events and the phenotypes of neurodegenerative diseases.^491^ TRF modulates the circadian rhythm to mitigate the Aβ load and impaired hippocampal transcription in AD models.^492^ Similarly, directly improving circadian rhythms activates various clock-controlled metabolic genes involved in insulin signalling and mitochondrial function and ameliorates Aβ pathology.^493^ Correspondingly, the management of circadian rhythms improves cognitive function and apathy in HD model mice.^494^ A disturbed circadian rhythm appears to be a frequent comorbidity of FTLD; thus, addressing sleep disturbances could improve the quality of life of patients.^495^

Additionally, CR induces autophagy to alleviate Aβ and tau pathologies and enhances cognitive function in AD models.^496^ CR also protects the survival of dopaminergic neurons in the substantia nigra, dopamine metabolism and neurotrophic factors in the striatum of PD animals.^497^ Similarly, abnormal eating behaviours, such as increased appetite and increased intake of sugar and carbohydrates, are universal in FTLD patients and correlate with atrophy in discrete neural networks,^498,499^ suggesting that dietary restriction may exert a positive effect.

Physical exercise engages a multitude of molecular mechanisms and should be prioritized for the elderly. Research has demonstrated that exercise can reduce inflammation levels, which is a contributing factor to the ageing process.^37^ High-resolution single-cell transcriptome sequencing has shown that long-term aerobic exercise effectively suppresses inflammation-related pathways and mitigates LPS-induced inflammatory responses across various organs in mice. Additionally, physical exercise plays a crucial role in protecting cardiovascular and respiratory functions,^490^ enhancing cardiorespiratory fitness by 20 to 40%, thereby correlating with observed reductions in cardiovascular events and all-cause mortality.^500^ Furthermore, exercise is also associated with the modulation of redox balance, age-related insulin resistance, and improvements in metabolic function.^501^ Another significant mechanism involves exercise-induced myokines that influence cell survival, neurogenesis, neuroinflammation, protein homoeostasis, oxidative stress, and protein modification.^502,503^ These beneficial changes underscore the importance of exercise in preventing and slowing the progression of ageing as well as neurodegenerative diseases.^504–508^ However, much more exploration into personalized, quantitative, and concrete lifestyle parameters, such as the type, duration and intensity of exercise, is needed.

Finally, we hypothesize that combining these positive lifestyles could eventually achieve better antiageing effects and manage neurodegenerative diseases.

## Antiageing strategies for neurodegenerative diseases: clinical trials

The current clinical trials for neurodegenerative diseases primarily focus on the molecular level in their antiageing strategies, while some studies also explore on the cellular and functional levels (Table 2).

**Table 2:** Clinical trials of the treatment of neurodegenerative diseases with antiageing agents

Classification	Drug	Identifier	Disease	Phase	Results	Ref.
Hormone supplementation or regulation	Allopregnanolone	NCT04838301	AD	Phase 2	Effective	^529^
	Allopregnanolone	NCT02221622	AD	Phase 1	Effective	^530^
	Isoflavones	NCT00205179	AD	Phase 2	Effective	^531^
Metabolic and nutritional regulation	Benfotiamine	NCT02292238	AD	Phase 2	Effective	^527^
	Caffeine	NCT04570085	AD	Phase 3		
	Fish oil Lipoic acid	NCT00090402	AD	Phase 1 Phase 2	Effective	^528^
	Glucagon-like peptide-1 agonists	NCT03659682	PD	Phase 2		
	Glucagon-like peptide-1 agonists	NCT04777409	AD	Phase 3		
	Insulin	NCT02503501	AD	Phase 2	Ineffective	^520^
	Metformin	NCT05781711	PD	Phase 2		
	Metformin	NCT04098666	AD	Phase 2 Phase 3		
	Rapamycin	NCT06022068	AD	Phase 1 Phase 2		
Anti-inflammation	Naproxen Sodium Celecoxib	NCT00007189	AD	Phase 3	Ineffective	^510^
	PTC857	NCT05349721	ALS	Phase 2		
Antiviral	Valacyclovir	NCT03282916	AD	Phase 2		^512^
Mitochondrial function regulation	Nilotinib	NCT03205488	PD	Phase 2	Ineffective	^518^
	Nilotinib	NCT02947893	PD	Phase 2	Lacks a placebo group and baseline differences: conclusions should be drawn cautiously	^519^
Synaptic modulation, anti-inflammation	CT1812	NCT03507790	AD	Phase 2		
	Simufilam	NCT05026177	AD	Phase 3		
Exercise	Aerobic exercise	NCT03808675	PD	Phase 2 Phase 3		^545^
	Aerobic exercises combined with dual-task training	NCT02074215	AD	Not applicable		

*AD* Alzheimer’s disease, *PD* Parkinson’s disease, *ALS* Amyotrophic lateral sclerosis

### Neuroprotection

This primarily encompasses: (1) Anti-inflammation or immune regulatory (including antibacterial or antiviral). The ageing process is characterised by an intensification of the inflammatory response and immunosenescence. The restoration of the body’s inflammatory balance, the regulation the response to endogenous and exogenous antigens of immune system could alleviate inflammation and the phenotype of neurodegenerative diseases, thereby promoting healthy ageing.^37^ For example, long-term, large-scale population trials on nonsteroidal anti-inflammatory drugs (NSAIDs) for AD treatment have shown that ibuprofen may have a protective effect on AD,^509^ while other NSAIDs seemingly do not.^510^ Ibuprofen has also been shown to reduce the brain age in elderly individuals by approximately one year.^511^ Antiviral drugs such as valacyclovir and some antibiotics are explored for AD treatment.^512^ (2) Antioxidant effects. The generation and elimination of free radicals are maintained in s dynamic equilibrium, which is essential for countering internal and external stimuli and preserving body’s internal environment homoeostasis. However, the oxidative stress during ageing process results in the destruction of the structure and function of intracellular macromolecules and organelles, resulting in cellular damage. Antioxidants mitigate oxidative reactions, protect cells from oxidative stress, and exhibit beneficial effects on neurodegeneration.^513^ Furthermore, antioxidants delay the ageing process of various organs/systems in elderly individuals, including the skin, ovaries, immune system, circulatory system, and brain.^514^ Drugs such as PYC857 are being studied for their antioxidant and anti-inflammatory effects on treating ALS. (3) Mitochondrial function regulation. Mitochondrial dysfunction arises a consequence of genomic instability, calcium ion overload, imbalanced redox reactions, dysregulation of mitochondrial turnover, and nutrient sensing pathways during the ageing process. Conversely, mitochondrial dysfunction can give rise to various ageing phenotypes through oxidative stress, activation of innate immunity, and cell apoptosis. Interventions targeting mitochondrial dysfunction have the potential to delay the process of ageing and neurodegenerative diseases.^515,516^ Nilotinib, proven to improve mitochondrial function,^517^ and clinical trials have been initiated to explore its application in PD.^518,519^

### Metabolic and nutritional regulation

The metabolism of glucose, lipids, proteins and vitamins in the body supplies energy and nutrients to the organism while maintaining physiological function. However, this process is disrupted during ageing, subsequently leading to the development of neurodegenerative diseases. Nutrient sensing networks sever as the fundamental mediator of cellular activities, and targeting these networks could potentially regulate the growth, development, and ageing process of organisms. Consequently, this offers a promising avenue for intervention in diseases.^85^ Moreover, drugs that target glucose and lipid metabolism, vitamins and other nutrients also exhibit neuroprotective functions, such as anti-inflammatory and antioxidant effects (e.g., the anti-inflammatory function of statins). These drugs include those targeting glucose metabolism, such as rapamycin, insulin, metformin, and GLP-1 RAs, for treatment of AD and PD.^520,521^ For example, a study randomized 38 AD patients to receive liraglutide or placebo and reported that liraglutide reversed AD-related glucose transporter dysfunction.^522^ Another single-blind, phase 2 trial evaluated exenatide, which improved motor deficits in PD patients for more than 12 months.^523^ Although the relationship between metformin and AD is contradictory, several clinical studies suggest that long-term metformin therapy is associated with a lower risk of neurodegenerative diseases.^524,525^ The first human clinical trial of NMN was conducted in 2021; NMN was administered to 25 older women, and the results revealed significant improvements in the muscle repair and regeneration capacity.^60^ Additionally, a phase 2 clinical trial in which a combination of metabolic activators (L-serine, N-acetyl cysteine, nicotinamide riboside, and L-carnitine tartrate) was used reported improved AD-related metabolic parameters and an approximately 20% increase in cognitive performance, although these benefits were not observed during the follow-up period.^526^ Additionally, vitamins or their derivatives, such as benfotiamine, as well as nutritional supplements, such as fish oil and caffeine, are also studied for AD treatment.^527,528^

### Hormone supplementation or regulation

Various hormones are closely linked to maintaining the normal function of the central nervous system. Hormonal dysregulation during ageing process impacts brain metabolism, synaptic plasticity, and cognitive function. It has been demonstrated that regulating hormone levels appropriately to adapt to age-related changes is advantageous in delaying age-related diseases.^349^ Clinical research on this mechanism shows promise. Age-related hormone disorders, such as a significant decrease in oestrogen levels in postmenopausal women, are associated with neurodegenerative diseases. Clinical studies on hormone supplementation include treatments such as allopregnanolone and isoflavones for AD.^529–531^ Other hormone-related treatments include GHRH supplementation in healthy elderly individuals and patients with mild cognitive impairment, and this treatment has favourable effects on cognition and metabolism.^532^ Previous studies have associated growth hormone administration for one year with an average reduction in BA of 2.5 years, as assessed by four epigenetic clocks.^533^

### Molecular replacement trials

The plasma of young individuals contains numerous antiageing factors that exert neuroprotective effects, such as anti-inflammatory and neurotrophic properties. Increasing the levels of these antiageing factors while replacing pro-ageing factors in older plasma through procedures like plasma exchange or young plasma infusion contribute to brain rejuvenation and the delay of neurodegenerative diseases.^201^ Therapeutic plasma exchange significantly rejuvenates the proteome and improves cognition in AD patients.^534,535^ AD patients who received four weekly infusions of young fresh frozen plasma showed promising outcomes, supporting further exploration of long-term plasma therapy.^73^

### Synaptic modulation and neural repair

During the process of ageing, impaired synaptic plasticity plays a significant role in neural ageing and age-related cognitive decline. By specifically targeting the deficits in impaired synaptic plasticity and directly modulating neuronal functions, it is possible to alleviate the phenotypes associated with ageing and neurodegenerative disease.^536^ Drugs such as CT1812 and simufilam are explored for AD treatment. Simufilam targets the altered form of filamin A, a scaffolding protein involved in several signalling pathways implicated in AD. By correcting altered filamin A levels, simufilam restores normal receptor signalling at synapses, improves synaptic function, reduces neuroinflammation, enhances synaptic integrity, and promotes cognitive function. CT1812 is a small molecule that displaces Aβ oligomers from synapses. These oligomers are toxic and disrupt synaptic function. By displacing them, CT1812 aims to restore normal synaptic function, potentially improving cognitive ability and slowing AD progression. CT1812 also reduces inflammation and promotes synaptic health, contributing to neural repair and cognitive restoration.

### Elimination of senescent cells

Cell senescence represents a pivotal event in the ageing process, whereby senescent cells secrete SASP to accelerate ageing of other cells and tissues. The elimination of senescent cells has been observed to mitigate ageing associated events, such as inflammation, stem cell exhaustion, and mitochondrial dysfunction.^537^ Additionally, clinical trials of senolytics, which selectively eliminate senescent cells and the SASP, have been conducted in patients with ageing-related diseases. Dasatinib plus quercetin (DQ) has been confirmed to improve physical dysfunction in 14 patients with idiopathic pulmonary fibrosis (IPF).^64^ Nevertheless, the roles of senolytics in neurodegenerative diseases need to be further validated. Inspiringly, a small-scale phase 1 clinical trial has shown that senolytics are safe, feasible and well tolerated in AD patients, and related phase 2 clinical trials are ongoing.^80^

### Lifestyle interventions

Ageing is a progressive decline of body’s function, whereas cognitive and behavioural training, exercise (such as dancing), and regulation of sleep and diet enhance overall physiological functioning by bolstering the body’s resilience to internal and external stimuli, promoting recovery in both the body and CNS, thereby ameliorating neurodegeneration.^538^ These therapies promote the rejuvenation of the body and central nervous system, ameliorating neurodegenerative diseases. (1) Physical training. Aerobic exercise reverses the age-related brain volume loss and physiological parameters in older adults.^539–541^ Moreover, aerobic exercise has been extensively researched for its role in improving cognition in AD patients and motor function in PD patients.^542–545^ (2) Sleep and diet. Regular sleep has been found to reduce the BA by up to 4.1 years.^546^ Inspiringly, first, from the Comprehensive Assessment of Long-term Effects of Reducing the Intake of Energy (CALERIE) trial, CR slows ageing in healthy adults by 2–3%, as measured by a DNA methylation biomarker for the pace of ageing calculated from the epigenome (DunedinPACE).^547^ CR also reduces the BA by 0.4 years.^548^ In addition, consuming a healthy diet, such as the Mediterranean-DASH (MIND) or Mediterranean diet, reduces age-related cognitive decline and decreases BA.^549^ Autopsy evidence indicates that MIND and Mediterranean diets are associated with less postmortem AD pathology, primarily a lower Aβ load.^550^ As expected, in a brief clinical trial, a multimodal intervention strategy combining diet, exercise and sleep resulted in an average reversal of the epigenetic age by 3.2 years.^551^

As mentioned above, antiageing treatments targeting different levels and mechanisms have become a hot topic in clinical research for neurodegenerative diseases. Some therapies have already proven effective, whereas others are actively being investigated. We eagerly anticipate the results of these ongoing studies and look forward to advancing further comprehensive treatments. In particular, the combination of antiageing therapies with monoclonal antibodies targeting pathological proteins holds promise for opening new avenues in the intervention of neurodegenerative diseases.

The primary objective of a comprehensive antiageing strategy is to halt or decelerate the progression of neurodegenerative diseases. Nevertheless, the current technological landscape can only achieve a limited degree of prevention by delaying or palliating the phenotypic manifestations associated with ageing and neurodegenerative disorders. Among various antiageing strategies, numerous studies have demonstrated their ability to delay or alleviate the hallmarks, pathological events, and physiological deficits linked to ageing and neurodegeneration in both animal models and patients with neurodegenerative diseases. Furthermore, additional antiageing strategies, such as anti-inflammatory medications and an active lifestyle (including a healthy diet, exercise, etc.), have also been shown to exert preventive effects by reducing the incidence of neurodegenerative diseases.

We present a synthesis of preclinical studies and clinical trials in holistic antiageing, which have significantly enhanced our understanding of the interplay between antiageing strategies and neurodegenerative diseases. However, numerous issues and challenges warrant further exploration. Firstly, these studies indicate the potential for delaying ageing or even partially reversing ageing-related phenotypes and disease manifestations. Nevertheless, the magnitude and duration of these effects require further investigation through long-term follow-up. Secondly, individual heterogeneity necessitates a comprehensive understanding of the underlying factors that influence antiageing outcomes across different individuals, including age, personality traits, comorbidities, lifestyle habits, education level, among others. Thirdly, while most studies have focused on the positive effects of these interventions, they often downplay side effects and adverse reactions, an aspect crucial for future clinical applications. Consequently, substantial work remains to be done in advancing future antiageing research. It is imperative to identify safe, effective targets for long-term antiageing interventions in neurodegenerative diseases. Additionally, it is vital to facilitating the transition from molecular mechanism investigations and animal models to clinical practice is vital.

## Conclusions and perspectives

With ageing, the body’s adaptive responses to stimuli decline and become insufficient to maintain dynamic homoeostasis, resulting in accumulation of pathogenic proteins (Aβ, hyperphosphorylated tau, α-syn and TDP-43) and neurodegeneration, further causing motor dysfunction and dementia. The integrated systems perspective aims to take a fresh look at the pathogenesis and treatment of neurodegenerative diseases: the development of neurodegenerative diseases is not necessarily traceable to a discrete molecular or cellular process but rather to the collapse of the interactions among many processes within and across organizational scales. These findings also provide novel perspectives on and opportunities for neurodegenerative disease research. Future studies on mechanisms should focus on finding upstream pathways for homoeostatic imbalances (pathogenic protein aggregation, neuronal degeneration and dysfunction of the organism) at different levels. Additionally, this theory may have implications for the diagnosis and early warning of neurodegenerative diseases. According to the ‘stimulus‒response’ model, intensifying the stimulus disrupts the balance, and the potential phenotype subsequently emerges. For example, the purpose of the exercise stress test is to increase the cardiac workload through a specific amount of physical activity, leading to electrocardiographic alterations in individuals with asymptomatic cardiovascular disease. For neurodegenerative diseases, a complex and multifactorial disease, restoring the body’s adaptability to stimuli and its ability to maintain homoeostasis may be more effective than simply removing pathological proteins. This change could be achieved by targeting multiple key nodes that have intervention effects on the whole system.

Current studies on the antiageing effects and prevention and treatment of neurodegenerative diseases are mostly conducted in artificially induced animal models, which differ from real changes in the human body. For example, many AD-related studies have been conducted in the classic APP/PS1 mouse model, to which the pathogenic gene causing familial AD has been transferred, subsequently inducing pathological events such as Aβ and hyperphosphorylated tau deposition. However, familial AD accounts for less than 5% of all cases of AD in patients. Therefore, APP/PS1 mice cannot imitate the pathogenesis of sporadic AD well, which is not conducive to subsequent research on mechanisms and treatments. Furthermore, these animal models may underestimate the contributions of peripheral and brain ageing to neurodegenerative diseases by inducing specific pathogenic events of neurodegenerative diseases through direct transgenization. Furthermore, the lifespan of mice, Drosophila, and nematodes is inadequate for accurately modelling the prolonged and gradual process of human ageing. Antiageing therapies should be validated in longer-lived species, such as non-human primates and naked mole rats. In these organisms, the molecular and functional changes associated with ageing that accumulate over time more closely resemble the human ageing phenotype, thereby providing a suitable foundation for research on ageing mechanisms and subsequent antiageing strategies. Therefore, the identification of more suitable animal models is urgently needed to simulate the process of neurodegenerative diseases during ageing.

As mentioned above, a potential disconnect exists between the healthspan and lifespan. A longer lifespan does not necessarily translate into a longer healthspan; in contrast, it may increase the burden of age-related neurodegenerative diseases. This finding suggests that the primary endpoint of research into the underlying mechanisms of rejuvenation should focus on health-related body parameters (e.g., liver function, renal function, metabolism and markers of ageing) rather than the mere lifespan. In addition, epidemiological studies should shift from traditional indicators (e.g., incidence and mortality) to DALYs, comprehensively and objectively evaluating the effectiveness of antiageing treatments in reducing the burden of neurodegenerative diseases and improving quality of life.

Ageing is a physiological process that progresses continuously. Throughout the ageing process, damage accumulates in the body as a result of its responses to internal and external changes, ultimately leading to dysfunction, chronic diseases, and death. The current objectives of antiageing research primarily focus on preventing, delaying, or mitigating the effects of ageing across all bodily systems; thus aiming to prevent the onset or delay the progression of neurodegenerative diseases. However, with advancements in antiageing research, targeting ageing for both prevention and treatment of neurodegenerative diseases will become increasingly feasible, ultimately enhancing healthy lifespan and overall quality of life. Despite the remarkable progress made in antiageing research, no single drug or approach is capable of exerting a comprehensive antiageing effect on humans. A multifaceted antiageing approach in which multiple targets at the same and different levels are intervented is recommended. For example, the combination of antiageing factors, drugs and an active lifestyle could be an effective strategy; however, the optimal combinations with minimal side effects remain to be determined. Consequently, this combination of antiageing modalities could be employed to prevent or delay the onset of neurodegenerative diseases, but further clinical trials are needed to substantiate this finding.

In conclusion, antiageing or rejuvenation interventions should be a critical step in combination with interventions targeting disease-specific events and comorbidities of neurodegenerative diseases; this strategy is promising for neurodegenerative disease therapy, and the implementation of comprehensive antiageing strategies that address the entire system is anticipated to yield enhanced efficacy. The future should prioritize efforts towards exploring methodologies for achieving comprehensive system rejuvenation, addressing disease-specific events and comorbidities associated with neurodegenerative diseases, and effectively translating these approaches into clinical practice.
